# Instability of PCN-224(Fe) during the oxygen reduction reaction; metal–organic framework electrocatalysts may have an Achilles heel

**DOI:** 10.1039/d6sc01428c

**Published:** 2026-04-10

**Authors:** Dana Rademaker, Roy Maas, Stefania Tanase, Hongrui Kang, Jan P. Hofmann, Dennis G. H. Hetterscheid

**Affiliations:** a Leiden Institute of Chemistry, Leiden University 2300 RA Leiden The Netherlands d.g.h.hetterscheid@chem.leidenuniv.nl; b Van ’t Hoff Institute for Molecular Sciences, Universiteit van Amsterdam 1098 XH Amsterdam The Netherlands; c Surface Science Laboratory, Department of Materials and Geosciences, Technical University of Darmstadt Peter-Grünberg-Straße 4 64287 Darmstadt Germany

## Abstract

Incorporation of catalytic sites within metal–organic frameworks has been considered advantageous as the amount of catalytic sites per cm^2^ electrode surface and be greatly expanded, while simultaneously the stability of the catalytic sties can be greatly improved. In contrast to previous studies, it is found that the dPCN-224(Fe) catalyst is extremely sensitive towards reactive oxygen species (ROS) formation during the electrochemical oxygen reduction reaction and deactivates within seconds. Very little is known about deactivation of catalytic sites in MOFs and what the effect could be on the overall catalytic performance of the MOF as a function of time. Herein, the degradation mechanism of dPCN-224(Fe) with ROS is studied in detail and it is shown that a few catalytic sites – presumably positioned at the interface between the MOF and the carbon support – are the specific sites targeted by ROS leading to the complete breakdown of all activity. Therefore, even though the density of active sites is exceptionally high in MOF systems, the catalytic reaction is strongly dependent on only few active sites that are directly positioned at the connection between the MOF and the carbon electrode or support.

## Abstract 2

Redox moieties that serve a dual role as electron transport hub and as a catalytic site represent a major stability concern in electrocatalysis featuring metal–organic frameworks.

## Graphical abstract

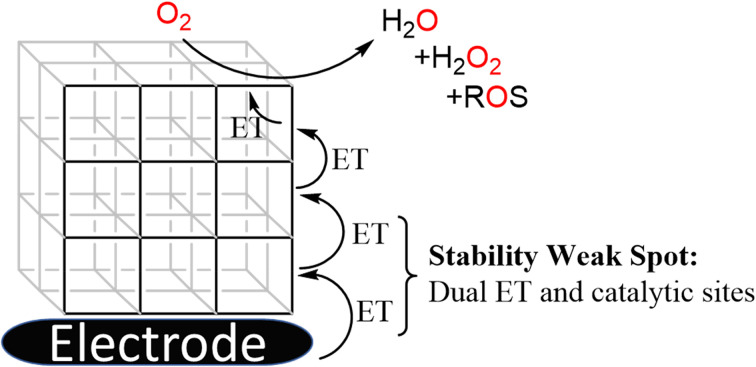

## Introduction

Metal–organic frameworks (MOFs) are three-dimensional structures made from inorganic nodes and (metal–)organic linkers. Due to the tunability and the porous structure of MOFs, they have gained attention as electrocatalyst for small molecule conversions.^1–7^ The porous structure allows for a large active surface area wherein well-defined catalytic sites are embedded at the node, linker, or as a guest directly at the working electrode of an electrochemical set-up.^8,9^

**Fig. 1:**
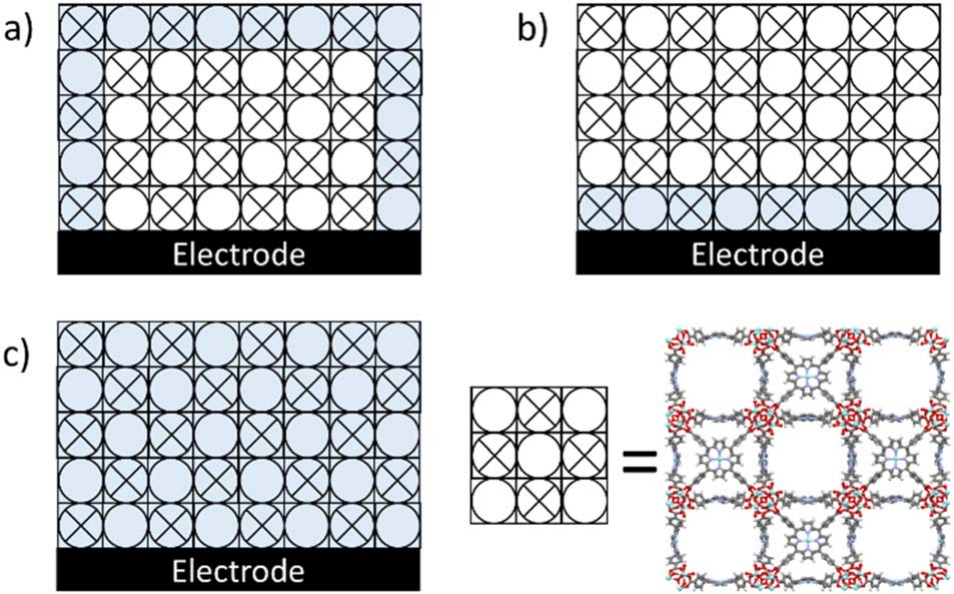
A cartoon of a PCN-224 MOF on an electrode with active sites participating in catalysis shaded in blue with the limiting factor of catalysis being (a) the diffusion of substrate, (b) the conduction of electrons and ions, and (c) the intrinsic catalytic activity of an individual active site. The crystallographic data was obtained from ref. 26.

Despite the potential of MOFs in catalysis thus far only a single application has made it to the market,^10^ which is mostly due to an unfavourable ratio between synthetic costs^11^ and MOF stability during catalytic performance. The stability of MOF-catalysts has predominantly been discussed in terms of the thermodynamics of linker–node interactions.^12–14^ Particularly high valent metal sites, such as Zr^IV^ or Ti^IV^, in combination with carboxylate functionalized linkers have been reported to be very robust.^15^ However, the +IV oxidation state in case of titanium and zirconium is very favourable, making these sites unreactive in redox catalysis or electron shuttling.^9^ Consequently additional redox sites and catalytic sites need to be implemented within such MOFs to mediate electrocatalysis.^16–18^ Whereas confinement effects to facilitate the activation of reactants is often discussed to improve the activity and/or selectivity of catalytic sites within MOFs,^19,20^ confinement effects have rarely been discussed as a trigger for catalyst degradation. The local confinement of reagents, pollutants^21^ and products may for example trigger significant local pH swings,^22^ or accumulation of other reactive species. To understand how MOF breakdown during electrocatalysis occurs it is important to consider where the catalytic activity takes place during electrocatalysis in MOFs. Where catalysis occurs precisely depends on the diffusion of substrate to the active site, the charge transfer of electrons and ions through the framework, and the intrinsic catalytic rate at these specific active sites.^23–25^ Depending on the limiting factor during catalysis, the number of active sites that participate in the catalytic reaction may therefore vary (Fig. 1). When the diffusion of the substrate is rate limiting, it is expected that catalysis will predominantly occur at the catalytic sites at the borders of a MOF particle. When the catalytic reaction is limited by charge transfer it is expected that mostly catalytic sites near the interface of the electrode will react. When the catalytic reaction is limited by the intrinsic catalytic rate at an individual site, it is expected that the catalytic reaction will occur more homogeneously throughout the entire MOF particle. Which of these factors is limiting depends on the precise reaction conditions, which includes the concentration of ions, the concentration of substrate, and the applied potential. At the start of the electrolysis the catalytic reaction likely starts at the interface of the MOF crystal and its support, given that the reactant is already loaded within the MOF, and then sweeps through the crystal when the substrate concentration is locally depleted. At these sites where catalysis occurs also damage to the MOF-structure may be expected.

**Fig. 2:**
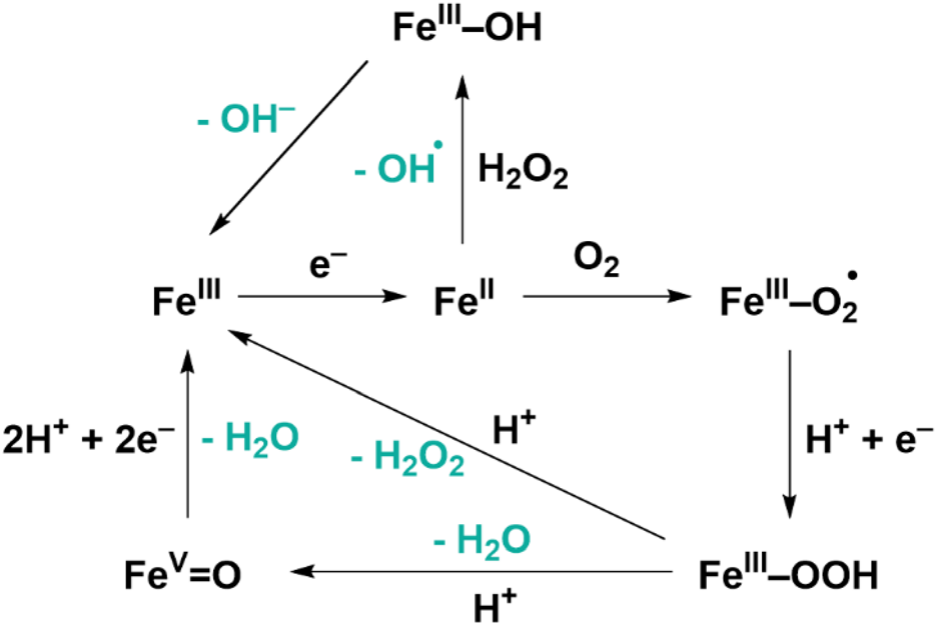
Mechanism of the ORR and Fenton reaction with iron porphyrin catalysts.^30,31^

We recently reported PCN-224(Co) with the cobalt 5,10,15,20-(4-carboxyphenyl)-porphyrin chloride (CoTCPP) as linker as an effective catalyst for the oxygen reduction reaction (ORR) with a high selectivity toward H_2_O_2_ of 80%.^27^ Moreover, the MOF was found to be highly stable upon build-up of H_2_O_2_ in the electrolyte solution. This high stability is an interesting finding as H_2_O_2_ is a strong oxidant that could lead to deactivation of catalytic sites if they were prone to oxidation by H_2_O_2_. The iron porphyrin-based MOFs PCN-224(Fe) and PCN-222(Fe) also showed to be active toward the electrochemical oxygen reduction reaction (ORR) under alkaline aqueous conditions and under acidic aqueous conditions, respectively.^28,29^ Iron 5,10,15,20-(4-carboxyphenyl)-porphyrin chloride (FeTCPP) is the active site in these porphyrin MOFs and during the ORR with this catalyst, the 2-electron ORR towards H_2_O_2_ formation is in competition with the 4-electron reduction towards H_2_O (Fig. 2).^30–32^ The H_2_O_2_ formed at the iron porphyrin sites can be activated *via* the Fenton reaction to form reactive oxygen species (ROS).^33–40^

Here it is found that the catalytic sites in the dPCN-224(Fe) catalyst are extremely sensitive under ORR conditions and deactivate within seconds. With the hypothesis in mind that MOF degradation pathways may be MOF specific due to the confinement of reactants, and local due to the catalytic reaction within the MOF not occurring uniformly (illustrated in Fig. 1) we studied the degradation mechanism of PCN-224(Fe) in the presence of O_2_ in detail. It is for the first time reported that a few catalytic sites that are presumably positioned at the interface between the MOF and the carbon support are the specific sites that are presumably targeted by ROS leading to the complete breakdown of all ORR activity.

## Results

**Fig. 3:**
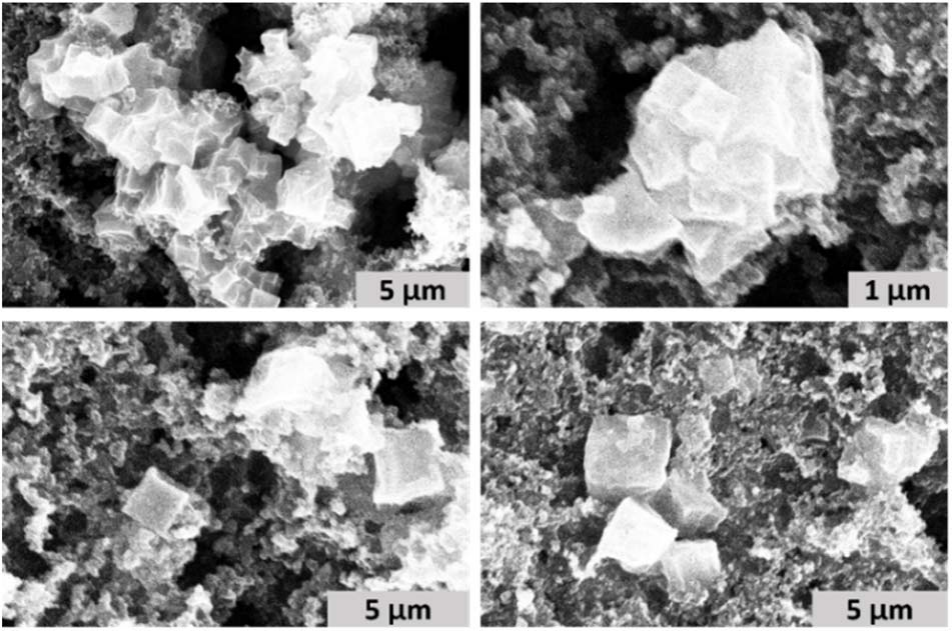
SEM image of PCN-224(Fe) ink measured at 15 kV and 0.1 nA.

PCN-224(Fe) was synthesized *via* a solvothermal synthesis by combining ZrCl_4_ and FeTCPP with benzoic acid as modulator in dimethylformamide as solvent.^41,42^ The framework that was formed was characterized by scanning electron microscopy (SEM), powder X-ray diffraction (pXRD), and N_2_-isotherm measurements. SEM images were collected from a drop of PCN-224(Fe) ink suspension, which contained PCN-224(Fe), carbon black, Nafion and acetone. The SEM images indicate a cubic morphology of the particles in the range of 1–4 µm, which agrees with the reported structure of PCN-224 (Fig. 3).^43^

**Fig. 4:**
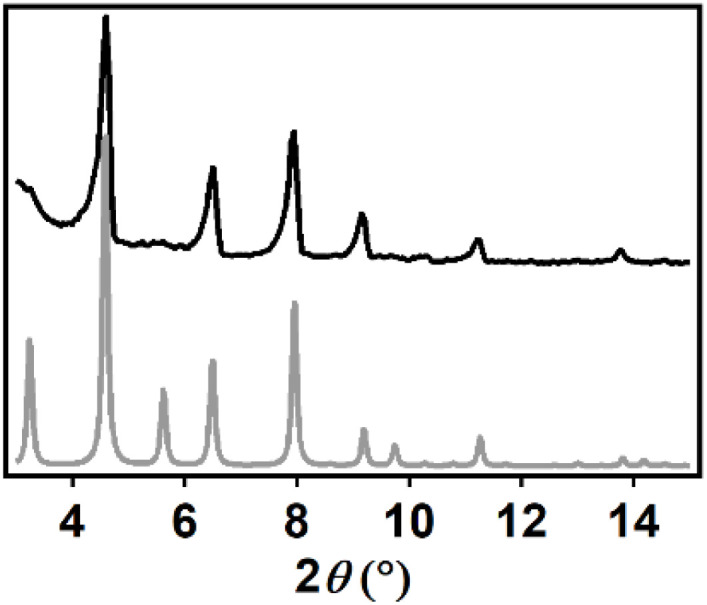
pXRD data of dPCN-224(Fe) synthesized in this work measured at 2° min^−1^ (black) and the theoretical diffractogram of PCN-224 (grey) that is based on the crystal structure of PCN-224 obtained by Zhou *et al.*^30^

The X-ray powder diffractogram matches the theoretical diffractogram of PCN-224, but lacks the reflections at 3.2 and 5.5° that indicate the presence of ordered PCN-224 superstructure domains in the MOF (Fig. 4).^44^ Therefore, the PCN-224(Fe) sample made in this work shows the reflections of a crystalline, but disorganized dPCN-224 structure.^43,44^ The MOF sample is therefore named dPCN-224(Fe). Furthermore, analysis of the N_2_-isotherm indicated pore sizes of 0.8 and 2.0–2.5 Å, as expected for PCN-224 (Fig. S1).^26^ Combined, these analytical data for the MOF sample are in good agreement with a dPCN-224 sample of good crystallinity.^43^

The ORR activity of dPCN-224(Fe) is compared to the activity of FeTCPP to investigate the role of the porous environment of dPCN-224(Fe) on the ORR activity, selectivity, and stability of the catalyst under ORR conditions. Rotating disk electrode (RDE) measurements are carried out in which the glassy carbon working electrode is modified with a dropcast containing the catalyst, carbon black (CB) as an electron conducting additive, and Nafion as an adhesive binder to maintain the physical intactness of the layer. This ink was dropcasted onto a glassy carbon electrode and allowed to dry in air. The ORR activity was assessed with RDE cyclic voltammograms (RDE CVs) in an aqueous solution containing 0.15 M HNO_3_ and 0.15 M NaNO_3_ under an oxygen atmosphere.

It is important to note that other electrolytes were used as well, including acetate buffer (pH 4.7), phosphate buffer (pH 7), and borate buffer (pH 8.5) and non-buffered Na_2_SO_4_ (pH 5), NaNO_3_ (pH 7), and NaOH (pH 14) electrolytes (SI 3). However, with all these electrolyte systems, the dPCN-224(Fe) MOF leached from the electrode during the first CV scan or directly when inserted into the electrolyte (Fig. S2). The dPCN-224(Fe) dropcast only remained intact in acidic electrolytes of HNO_3_, H_2_SO_4_ or HClO_4_. Whereas other PCN-224(Fe) materials have been described to show good ORR activity in alkaline 0.1 M electrolyte solutions,^28^ the cubic PCN-224(Fe) crystals obtained in this study are not stable in alkaline media and dissolve immediately.

**Fig. 5:**
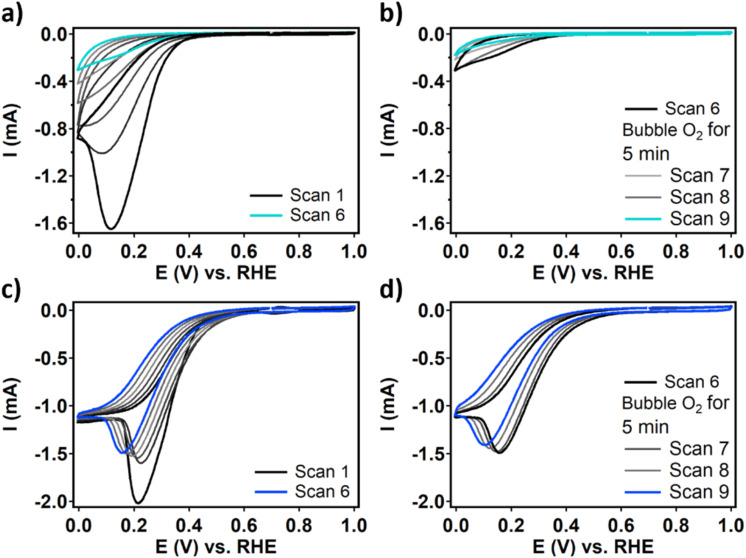
Six RDE CV scans of (a) dPCN-224(Fe) and (b) three RDE CV scans after bubbling oxygen for 5 minutes. Six RDE CV scans of (c) FeTCPP and (d) three RDE CV scans after bubbling oxygen for 5 minutes. Measured in 0.15 M HNO_3_ and 0.15 M NaNO_3_ with 50 mV s^−1^ scan rate and 1600 rpm rotation rate under an oxygen atmosphere.

RDE CV measurements of dPCN-224(Fe) and FeTCPP show that in the first scan both catalysts are active for the ORR resulting in large peak-shaped curves (Fig. 5 and SI 4). The shapes of these curves are unusual for RDE experiments and indicate that an irreversible event is occurring during the ORR that is not mass transport limited in oxygen. Moreover, for dPCN-224(Fe) the current collapses upon the continuation of the CV experiment and after nine scans, the ORR activity is completely lost (Fig. 5b). This behavior was also found for a sample of phase-pure PCN-224(Fe) in acidic aqueous electrolyte, which indicates that the absence of the long-range superstructure in dPCN-224(Fe) is not the cause of the decreasing ORR current (Fig. S6). For FeTCPP, the change in the CV upon prolonged scanning is more gradual. The maximum current decreases while the catalytic wave shifts cathodically (Fig. 5c). This RDE CV shows a plateau current with a cathodic peak at 0.25 V *vs.* RHE on top of the plateau. This additional cathodic peak might be indicative of a mismatch between the elementary steps during ORR catalysis.^45–47^ If the first activation of the Fe superoxide intermediate *via* a proton and electron transfer is limiting catalysis, the Fe superoxide species might accumulate before it reacts toward the Fe hydroperoxo intermediate at a higher overpotential. If the activation of this Fe hydroperoxo species is limiting, H_2_O_2_ might form locally which is further reduced to H_2_O at a higher potential.

**Fig. 6:**
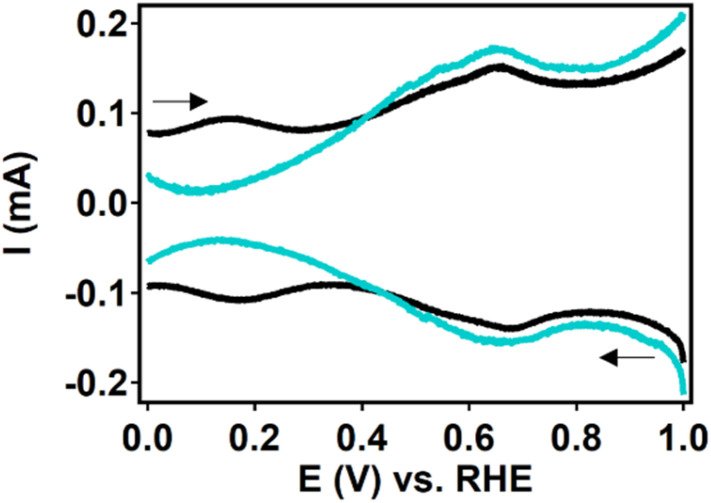
DPV measurement of dPCN-224(Fe) before (black) and after (teal) ORR electrocatalysis. Measured in 0.15 M HNO_3_ and 0.15 M NaNO_3_ with 2 mV step size, 50 mV modulation amplitude, 3 ms modulation time and 50 ms interval time under an argon atmosphere.

During the ORR, the iron species is reduced from Fe^III^ to Fe^II^ before oxygen binds (Fig. 2). This redox couple can be visualized by measuring a differential pulse voltammetry (DPV) experiment in the absence of oxygen. Therefore, a DPV measurement was carried out under argon atmosphere with a fresh dropcast containing dPCN-224(Fe) (Fig. 6). In the DPV trace two redox couples can be distinguished at 0.65 V *vs.* RHE and at 0.17 V *vs.* RHE. The peak at 0.65 V *vs.* RHE was also observed for PCN-224(H_2_), which is the same MOF without the iron center coordinated in the porphyrin pocket (Fig. S7). Broad peaks at around 0.6–0.7 V *vs.* NHE have previously been associated with proton-coupled electron transfers of quinone functionalities on a carbon support.^48,49^ Therefore, this peak is not expected to be related to the iron porphyrin complex. The peak at 0.17 V *vs.* RHE is assigned to the Fe^II^/Fe^III^ couple.^50^ After measuring DPV under argon atmosphere, the dPCN-224(Fe) sample was subjected to an RDE CV measurement of nine scans under oxygen atmosphere, after which DPV was recorded again under argon atmosphere (Fig. 6). The DPV after electrocatalysis reveals that the redox couple at 0.17 V *vs.* RHE is no longer present.

From reports about photooxidation with dPCN-224(Fe) it is known that H_2_O_2_ and reactive oxygen species (ROS) can induce degradation of the catalyst.^33–37^ To verify if H_2_O_2_ also plays a role in the irreversible diminishing of the ORR current for dPCN-224(Fe), the ORR selectivity is investigated. During the ORR, the two-electron oxygen reduction reaction (*E*° = 0.695 V *vs.* NHE) towards H_2_O_2_ is in competition with the four-electron oxygen reduction reaction towards H_2_O (*E*° = 1.23 V *vs.* NHE). The faradaic efficiency of the ORR towards H_2_O_2_ (% H_2_O_2_) with dPCN-224(Fe) or FeTCPP is investigated with rotating rink disk electrode linear sweep voltammetry (RRDE LSV) experiments (SI 7). To determine % H_2_O_2_ the average of three measurements was used. dPCN-224(Fe) shows a % H_2_O_2_ of ∼19 ± 5% over the whole potential range, while FeTCPP shows a % H_2_O_2_ of ∼32 ± 7% at 0.4 V *vs.* RHE and ∼8 ± 2% at 0.15 V *vs.* RHE. These results indicate that during the ORR with dPCN-224(Fe) and FeTCPP H_2_O_2_ is indeed being formed.

Reactive oxygen species (ROS) such as the hydroxyl radical can be formed by the Fenton reaction catalyzed by an iron species:^51^1
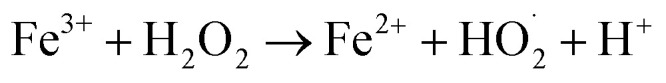

2 Fe^2+^ + H_2_O_2_ → Fe^3+^ + HO˙ + OH^−^

**Fig. 7:**
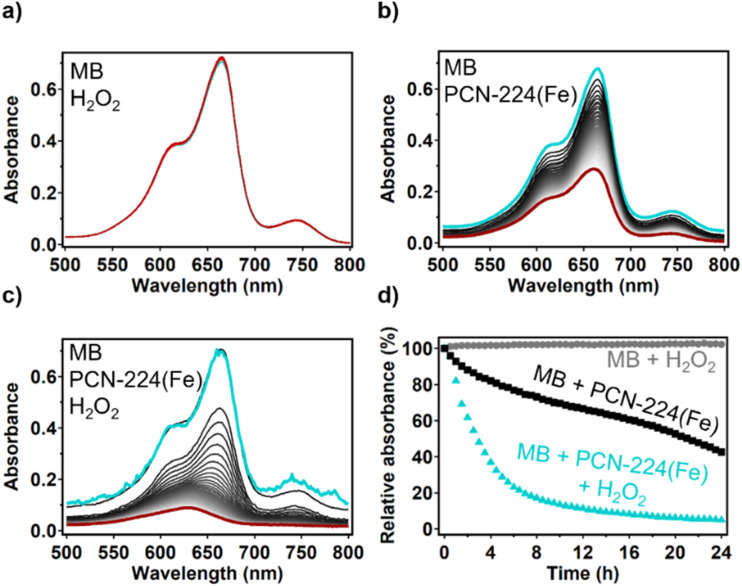
UV-vis spectra with (a) MB and H_2_O_2_, (b) dPCN-224(Fe) and MB, and (c) dPCN-224(Fe), MB and H_2_O_2_ measured every 30 minutes for 24 h. The spectrum at *t* = 0 is shown in teal and the curve at *t* = 24 h is shown in red. (d) The relative absorbance at 665 nm over time under the three different conditions: [MB + H_2_O_2_] in grey, [dPCN-224(Fe) + MB] in black, and [dPCN-224(Fe) + MB + H_2_O_2_] in teal. Concentrations: 6.0 µg mL^−1^ MB, 10 mM H_2_O_2_, and 0.17 mg mL^−1^ PCN-224(Fe) with a total cuvette volume of 3 mL.

The possibility of an iron species to carry out the Fenton reaction depends on the nature of the iron species and the electrolyte.^52^ An extensive study was carried out by Yang *et al.* in which the Fenton reaction catalyzed by FeTCPP was investigated.^53^ The FeTCPP catalyst was used to degrade the organic molecule bisphenol A in the pH range of 4–12. *Via* 5,5-dimethyl-1-pyrroline *N*-oxide (DMPO) trapping experiments, hydroxide radicals could be found in the reaction mixture. This indicates that the porphyrin site FeTCPP that is present in dPCN-224(Fe) is capable of Fenton chemistry. The ability of dPCN-224(Fe) to generate hydroxyl radicals from H_2_O_2_ was tested by combining the MOF with H_2_O_2_ and the organic dye methylene blue in 0.15 M HNO_3_ and 0.15 M NaNO_3_. Methylene blue (MB) is an organic dye that is specifically degraded by hydroxide radicals as shown by radical trapping experiments with DMPO in previous reports.^54–56^ The degradation of the MB dye can be monitored with UV-vis spectroscopy. The UV-vis signal of MB was found to be stable after addition of H_2_O_2_ to the acidic MB solution (Fig. 7a), which indicates that H_2_O_2_ itself cannot degrade MB. Combining the dPCN-224(Fe) MOF with the acidic MB solution resulted in 57% loss of MB signal after 24 h (Fig. 7b). This loss of the UV-vis signal of MB can be explained by absorption of the organic dye in the pores of the MOF, which was also observed with PCN-224(Co) (Fig. S10) and the iron-based MOF NH_2_-MIL-88B(Fe).^56^ Upon addition of both the dPCN-224(Fe) and H_2_O_2_ to the acidic MB solution, a more rapid decrease of the MB signal is seen and 95% of the UV-vis signal at 665 nm is lost after 24 h (Fig. 7c and d). This large decrease of the UV-vis signal of MB indicates that dPCN-224(Fe) can indeed generate hydroxyl radicals from H_2_O_2_ that degrade the MB dye. Moreover, upon addition of H_2_O_2_ to dPCN-224(Fe) the formation of bubbles was observed, which indicates that the MOF can also catalyse the disproportionation of H_2_O_2_ to water and oxygen (Fig. S11). The activation of H_2_O_2_ by dPCN-224(Fe) is an interesting finding, since iron mostly has a Fe(iii) oxidation state in this MOF, while it is Fe(ii) that is expected to mostly accelerate Fenton chemistry *via* the reductive pathway. This leads to a slow activation of H_2_O_2_ in the UV-vis experiment. During the electrochemical ORR, the iron sites are expected to be reduced to Fe(ii) by the electrode material, which makes the iron sites more active for the Fenton reaction and fast decomposition of H_2_O_2_ to hydroxide radicals is expected.

**Fig. 8:**
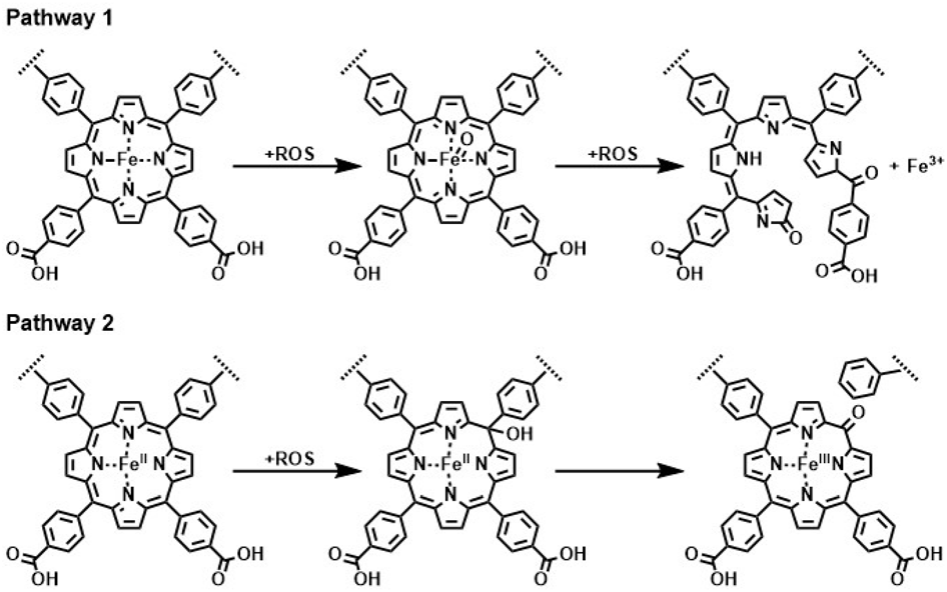
Reported degradation pathways of an iron porphyrin of the dPCN-224(Fe) MOF upon contact with a reactive oxygen species being either H_2_O_2_ or a radical species.^39,57–59^

It was shown that dPCN-224(Fe) produces H_2_O_2_ during the electrochemical ORR and that dPCN-224(Fe) can degrade H_2_O_2_ into hydroxyl radicals. Therefore, the degradation of dPCN-224(Fe) might be caused by interactions of these radical species with the porphyrin linkers of the MOF. The interactions of ROS with iron porphyrins have been investigated previously.^58,60–64^ In nature, iron porphyrins of peroxidases are often damaged by the reaction of the heme with H_2_O_2_.^60^ In this peroxidase, porphyrin degradation was found to proceed *via* ring-cleavage oxidation at the *meso*-position of the porphyrin. This porphyrin degradation mechanism *via* ring-opening with loss of iron ions was also found for dPCN-224(Fe) upon H_2_O_2_ treatment under UV-light irradiation (Fig. 8).^39^ Furthermore, degradation of an iron porphyrin in pyridine solution was found to occur due to attack by ROS and a degradation pathway in which the *meso*-position is oxidized with retention of the N_4_-binding pocket of the porphyrin was identified (Fig. 8).^57,58^ This pathway was also reported as possible degradation pathway of heme in nature, in which *meso*-hydroxyheme is formed upon interaction with hydrogen peroxide.^59^ To understand what happens to dPCN-224(Fe) during the ORR, the structure and chemistry of dPCN-224(Fe) before and after catalysis was assessed with SEM, pXRD, inductively coupled plasma mass spectrometry (ICP-MS), X-ray photoelectron spectroscopy (XPS), solid state UV-vis, and Fourier-transform infrared spectroscopy (FTIR) measurements.

**Fig. 9:**
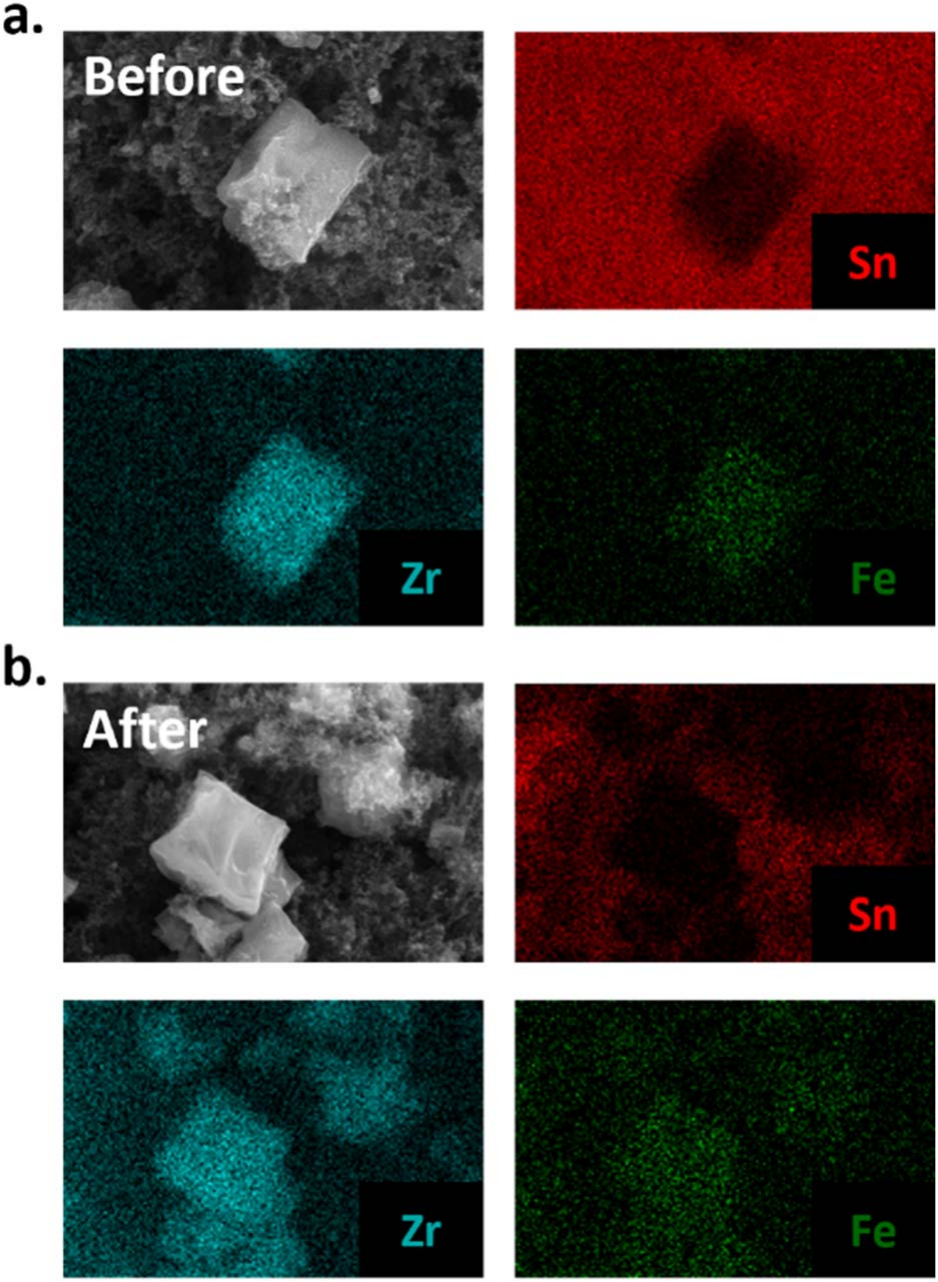
SEM-EDX of dPCN-224(Fe) (a) before and (b) after 20 CV cycles. Elemental mapping is given for Sn (red), Zr (teal) and Fe (green).

**Fig. 10:**
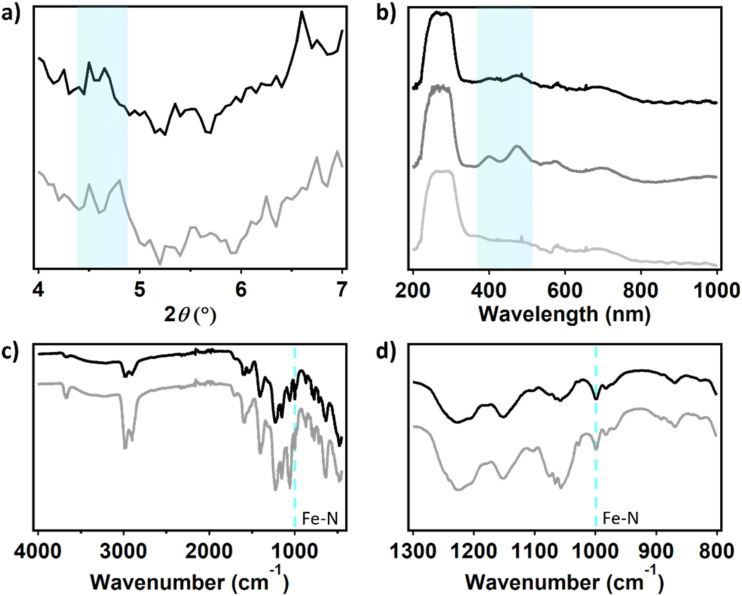
(a) pXRD before (grey) and after (black) catalysis of dPCN-224(Fe) ink on an FTO electrode. (b) UV-vis of bare FTO (light grey), FTO with dPCN-224(Fe) fresh after dropcast (dark grey) and FTO with dPCN-224(Fe) after electrocatalysis (black). (c) FTIR of FTO with dPCN-224(Fe) fresh after dropcast (grey) and FTO with dPCN-224(Fe) after electrocatalysis (black) and (d) FTIR spectrum zoomed in between 1300 and 800 cm^−1^.

To evaluate the morphology of the dPCN-224(Fe) particles before and after ORR catalysis, a fluorine doped tin oxide (FTO) electrode was covered with the catalyst ink solution and used for twenty CV cycles under oxygen atmosphere. During these 20 CV scans the activity was completely lost (Fig. S12). After drying of the electrode, SEM images were collected of a fresh dropcast sample and a sample that was subjected to catalysis (Fig. S13). The size and shape of the cubic particles are retained after catalysis. Moreover, elemental mapping with SEM-EDX (EDX = energy dispersive X-ray) measurements were carried out to identify the FTO background with Sn and the MOF particles with Zr and Fe contents (Fig. 9). The elemental mapping indicates the presence of Zr and Fe at the position of the MOF particles before and after catalysis as well as Sn at the places without MOF particles. This indicates that the iron is not removed from the framework during catalysis. This was confirmed with ICP-MS analysis of dPCN-224(Fe) by dissolving a fresh dropcast of an FTO electrode and a dropcast of an FTO electrode used for catalysis in HNO_3_ solution. In the fresh sample 2.09 ± 0.01 Fe ions per Zr_6_ node were found, while the used samples showed 2.02 ± 0.10 Fe centers per node (SI 11). Moreover, XPS analysis of the dPCN-224(Fe) samples before and after catalysis illustrate that the MOF is retained during catalysis. Substantial amounts of Zr can be detected in samples before and after catalysis. However, Fe, due to its low surface concentration, could not be detected before and after catalysis. Also, the nitrogen content was too low to make a conclusive statement on the state of N from the N 1s spectra. This may suggest that the outside of the MOF particles predominantly consist of Zr-nodes. pXRD measurements were carried out with the FTO electrode covered with the dPCN-224(Fe) ink to investigate the porosity of the framework after catalysis (Fig. 10a). An FTO electrode was covered with 50 µL ink and used for 20 CV cycles under oxygen atmosphere (Fig. S15). pXRD was measured of a fresh FTO electrode with dPCN-224(Fe) ink on it and of an FTO electrode used for electrocatalysis (Fig. 10a). To obtain a dry sample after catalysis, the FTO plate with dropcast was soaked in water to remove electrolyte ions and in DCM to remove the water molecules and allow for drying. During these soaking cycles, some of the dropcast detached from the FTO electrode. Therefore, there are less MOF particles after catalysis than in a fresh dropcast, which leads to lower intensity signals in characterization methods used herein. Nevertheless, the pXRD indicates faint reflections at 4.6° for both FTO electrodes, which indicates the porosity is maintained after catalysis (Fig. 10a).

Solid state UV-vis was carried out to investigate the porphyrin UV-vis absorbance of dPCN-224(Fe) before and after catalysis on an FTO electrode (Fig. 10b). A dPCN-224(Fe) ink was made without CB, because the carbon would absorb all the light. The FTO electrode was used for 20 CV cycles under oxygen atmosphere (Fig. S18). A bare FTO electrode, fresh dropcast on FTO and the dropcast used for electrocatalysis were investigated with solid state UV-vis spectroscopy. The fresh dropcast shows two absorbance peaks at 399 and 473 nm that are not present for the bare FTO sample and originate from the porphyrin structure in dPCN-224(Fe). For FeTCPP a Soret band at 419 nm and a Q-band at 535 nm have been documented previously.^65,66^ Therefore, for dPCN-224(Fe) the absorption peaks are assigned to a Soret band at 399 nm and a Q-band at 473 nm, which is blue-shifted compared to FeTCPP itself. These absorption peaks are still present in the dropcast after catalysis, which indicates that the porphyrin structure remains intact.

FTIR was carried out with the same FTO electrodes without CB to investigate the bonds present in the dPCN-224(Fe) framework before and after electrocatalysis (Fig. 10c). All peaks of the fresh sample are found back in the sample after catalysis and are assigned in Table S3. Moreover, the vibration at 999 cm^−1^ of Fe–N is present in both spectra, which is indicative of the retention of Fe in the N_4_-pocket of the porphyrin (Fig. 10d).^35^ Based on the SEM-EDX, ICP-MS, XPS, pXRD, solid-state UV-vis, and FTIR measurements, it can be concluded that no significant change of the porphyrin and Zr node content has taken place after ORR catalysis with dPCN-224(Fe).

## Discussion

**Fig. 11:**
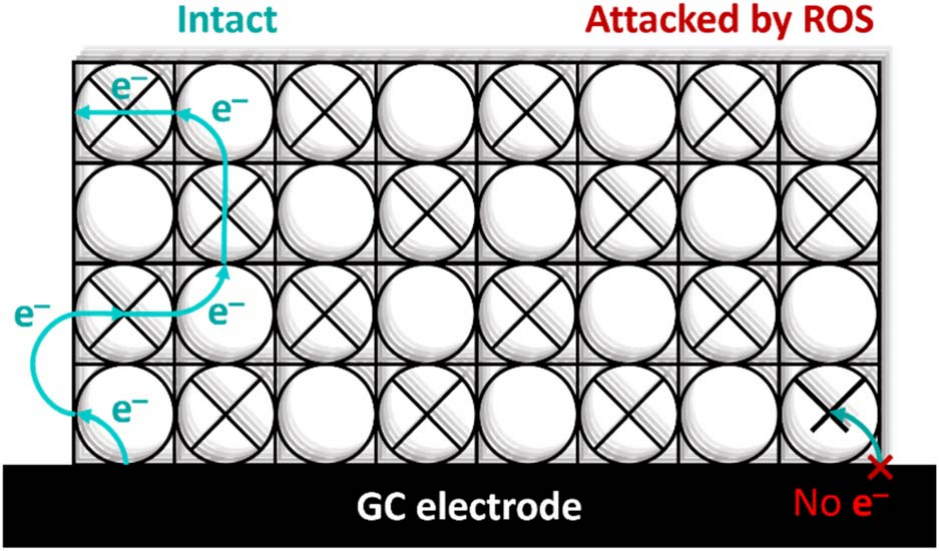
Schematic overview of electron conduction in dPCN-224(Fe) when intact or when the porphyrin site near the glassy carbon (GC) working electrode is inactive due to degradation.

For dPCN-224(Fe) the ORR activity is completely lost after nine consecutive CV cycles in oxygen environment. This is in contrast to the PCN-224(Co),^27^ which is stable in presence of H_2_O_2_, thereby linking the observations in case of PCN-224(Fe) to the presence of iron. The analysis of the dPCN-224(Fe) structure before and after catalysis with SEM, pXRD, ICP-MS, solid state UV-vis, and FTIR measurements were the same before and after catalysis, which entails that the bulk of the MOF remains unchanged after catalysis. This indicates that the complete shutdown of catalysis is not due to a complete destruction of the MOF particles. Interestingly, DPV measurements indicate the complete disappearance of the Fe^II^/Fe^III^ couple after ORR catalysis, which indicates that there is no longer an electrochemical connection with Fe-sites in the MOF. The complete shutdown of catalysis, the disappearance of the Fe^II^/Fe^III^ redox couple, and the retention of the bulk structure of the MOF points to key sites within the MOF framework to be decomposed. These sites could either have a role as a catalytic site or as an electron transfer site. Given that experiments with Fe-TCPP under the same conditions, and many other iron porphyrin systems reported in literature that do not rely on an electron transport chain,^67–70^ do not breakdown in a spectacular manner under ORR conditions, irrespective of the presence of H_2_O_2_ and ROS generation, illustrates that the rapid breakdown of all catalytic activity in PCN-224(Fe) is an anomaly. The injection of the first electron from the electrode material to the MOF occurs at the Fe(iii) sites that are closest to the electrode support. Electron conduction in MOFs with spatially separated electroactive linkers, such as dPCN-224(Fe), occurs *via* hopping between the porphyrin sites.^71–73^ So upon reduction of this first critical Fe(iii) site next to the electrode material to an Fe(ii) species, the electron can be further transferred to an Fe(iii) site in the vicinity. This electron hopping method then continues *via* a diffusion-like hopping mechanism through the framework.^23,74^ This electron hopping mechanism is thus dependent on the first electron transfer between the electrode material and the MOF particle. Upon this first electron transfer, the formed Fe(ii) species next to the electrode interface can either continue with the electron hopping or bind oxygen to perform the ORR. During the ORR, H_2_O_2_ is formed which can react with Fe(ii) to form ROS *via* the Fenton reaction and trigger decomposition.^24,75^ When these critical sites of the framework near the electrode are damaged, the electron conduction pathway is impeded and catalysis will be completely halted (Fig. 11), which would be in line with the severely more devastating collapse of catalytic activity in PCN-224(Fe) compared to Fe-TCPP and other Fe-porphyrin systems.

## Conclusions

From this work it can be concluded that changing the metal in the porphyrin pocket of the PCN-224 MOF from cobalt to iron can drastically change the stability, selectivity, and activity of the ORR electrocatalyst. Even though the majority of the PCN-224(Fe) MOF is perfectly intact, all electronic communication between the carbon support and the MOF is lost after a short burst of electrocatalysis. This suggest, that even though the density of active sites is exceptionally high in MOF systems, the catalytic reaction is strongly dependent on the stability of only few active sites that are involved in the electron transfer chain. The dual role of the iron sites in electron transport and catalysis, and the stability issues associated, represent a major hurdle in the development of MOF systems as stable electrocatalysts.

## Author contributions

DR: investigation, writing – original draft, writing – review and editing; RM: investigation, writing – original draft; ST: resources; HK: formal analysis; JPH: formal analysis, supervision, resources; DGHH: conceptualization, funding acquisition, supervision, writing – review and editing.

## Conflicts of interest

There are no conflicts to declare.

## Supplementary Material

SC-OLF-D6SC01428C-s001

## Data Availability

The data supporting this article have been included as part of the supplementary information (SI). Supplementary information: N_2_ isotherm, chemical stability dPCN-224(Fe), triplo RDE CV dPCN-224(Fe) and FeTCPP, RDE CV with PCN-224(Fe), DPV PCN-224(H_2_), triplo RRDE LSV with dPCN-224(Fe) and FeTCPP, UV-vis study with PCN-224(Co), bubble formation dPCN-224(Fe) and H_2_O_2_, SEM dPCN-224(Fe), ICP-MS dPCN-224(Fe), XPS dPCN-224(Fe), FTIR dPCN-224(Fe). See DOI: https://doi.org/10.1039/d6sc01428c.
