# Supplementary material for: Instability of PCN-224(Fe) during the oxygen reduction reaction; metal–organic framework electrocatalysts may have an Achilles heel
Source: Chem Sci. 2026 Apr 10;17(24):11932–40. doi: 10.1039/d6sc01428c (PMC13158803; doi:10.1039/d6sc01428c)
Supplement: SC-017-D6SC01428C-s001 [file SC-017-D6SC01428C-s001.pdf]

Supplementary Information (SI) for Chemical Science.
This journal is © The Royal Society of Chemistry 2026

Supporting information

Instability of PCN-224(Fe) during the Oxygen Reduction Reaction; Metal-Organic Framework Electrocatalysts may have an Achilles heel

Dana Rademaker,[a] Roy Maas,[a] Stefania Tanase,[b] Hongrui Kang,[c] Jan P. Hofmann,[c] and Dennis G.H. Hetterscheid[a]

[a] Leiden Institute of Chemistry, Leiden University, 2300 RA Leiden, The Netherlands.
E-mail: d.g.h.hetterscheid@chem.leidenuniv.nl.

[b] Van 't Hoff Institute for Molecular Sciences, Universiteit van Amsterdam, 1098 XH Amsterdam, The Netherlands.

[c] Surface Science Laboratory, Department of Materials- and Geosciences, Technical University of Darmstadt, Peter-Grünberg-Straße 4, 64287 Darmstadt, Germany.

Contents

1. Experimental details 3
1.1 Materials and methods 3
1.2 Synthesis protocols 3
5,10,15,20-(4-carboxyphenyl)-porphyrin (TCPP) 3
Fe(5,10,15,20-(4-carboxyphenyl)-porphyrin)chloride (FeTCPP) 3
dPCN-224(Fe) 3
1.3 Electrochemistry 4
Electrochemical set-up 4
Catalyst ink preparation 4
Rotating (ring) disk electrode cyclic voltammetry 4
2. N_2-isotherm *d*PCN-224(Fe) 6
3. PCN-224(Fe) chemical stability in electrolyte solution 7
4. RDE CV with *d*PCN-224(Fe) and FeTCPP 8
5. ORR with PCN-224(Fe) 9
6. DPV PCN-224(H_2) 10
7. RRDE LSV with *d*PCN-224(Fe) and FeTCPP 11
8. UV-Vis study with PCN-224(Co) and methylene blue 12
9. UV-Vis study with *d*PCN-224(Fe) and methylene blue 13
10. SEM *d*PCN-224(Fe) 14
11. ICP-MS PCN-224(Fe) 15
12. XPS of *d*PCN-224(Fe) 16
13. FTIR of *d*PCN-224(Fe) 17
14. References 19

1. Experimental details

1.1 Materials and methods

For synthesis, methyl 4-formylbenzoate (Sigma Aldrich, 99%), propionic acid (Alfa Aesar, 99%), pyrrole (Sigma Aldrich, >98%), DMF (VWR), THF (VWR), methanol (VWR), KOH-pellets (Thermo Fisher, 85%), HCl (37%, VWR), $FeCl_2$•$4H_2O$ (Thermo Scientific Chemicals, > 99%), ZrCl4 (Alfa Aesar, >99.5%), benzoic acid (Alfa Aesar, >99.5%), H_2O_2 (30% w/w, Sigma Aldrich), and methylene blue (Alfa Aesar) were used without further purification. Milli-Q Ultrapure grade water (>18.2 MΩ cm resistivity) was used for all experiments. $ZrCl_4$ was stored in a desiccator. The H_2O content in DMF was regularly monitored by a Karl-Fischer titration and was found to be between 1000 and 3300 ppm.

NMR data was recorded on a Bruker Advance 400/101 MHz Ultrashield NMR Spectrometer using residual solvent as an internal standard. Elemental analysis was performed by Mikroanalytisches Laboratorium Kolbe in Oberhausen, Germany. The water content of the DMF was quantified by using a TitroLine 7500 KF trace titrator. Powder X-ray diffraction (pXRD) spectra were measured on a Rigaku Miniflex II desktop X-ray diffractometer with Cu Kα radiation (λ=1.5406 Å) with 0.05° steps and a scan speed of 2°/min. Scanning electron microscopy (SEM) images were collected with a Thermo Scientific Apreo scanning electron microscope operating under a high vacuum. Data for images were recorded at an accelerating voltage of 15 kV with a 0.2 nA probe current. SEM samples were prepared by depositing a 5 nm layer of platinum on top of the sample using a Cressington 208 HR sputter coater. Elemental mapping was carried out with a UltraDry energy dispersive X-ray (EDX) detector at an accelerating voltage of 20 kV with a 1.6 nA probe current. The N_2-adsorption isotherm was measured on a Belsorp II max and data was analyzed with BELMaster software. UV-Vis data was recorded on an Agilent Varian Cary 50 spectrophotometer. Solid state UV-Vis was recorded with a reflection probe on an Avantes AvaSpec–2048 spectrometer and using an Avalight–DH–S-Bal light source with $BaSO_4$ as the reflectance standard reference. FTIR spectra were recorded on a Spectrum Two FT-IR spectrometer from Perkin Elmer. ICP-MS was measured on a NexION® 2000 ICP Mass Spectrometer from Perkin Elmer. X-ray photoelectron spectroscopy (XPS) data were obtained on a Physical Electronics PHI Versaprobe 5000 spectrometer in fixed analyzer transmission mode using monochromatic Al Kα radiation (hν = 1486.6 eV, spot diameter 200 μm and a power of 50 W) at an analyzer angle of 45° with a pass energy of 23.50 eV (step size of 0.1 eV) for region scans and survey scans over a range of 1350.0 to -10.0 eV with a step size of 0.8 eV and a pass energy of 187.850 eV. The samples dropcasted on FTO electrodes have been measured as received; no precautions to avoid air exposure during sample handling have been taken. A charge neutralizer equipped with a low-voltage electron gun was used to compensate for sample charging. Elemental compositions were determined using Scofield relative sensitivity factors available in CasaXPS 2.3.23PR1.0.

1.2 Synthesis protocols

5,10,15,20-(4-carboxyphenyl)-porphyrin (TCPP)

5,10,15,20-(4-carboxyphenyl)-porphyrin was synthesized according to a reported procedure.[1] Methyl-4-formylbenzoate (10.9 g; 66.4 mmol) was dissolved in propionic acid (250 ml). While stirring this clear solution, pyrrole (5 ml; 72.1 mmol) was added. The brown solution was refluxed for 1.5 h, after which the mixture was allowed to cool to r.t. The mixture was filtered and washed with acetone (5 × 15 mL). After drying, the purple solid was dissolved in a solution of 1:1:1 of THF:methanol:1.9 M KOH in water (225 mL) and this brown solution was refluxed overnight while stirring. The brown solution was acidified by slow addition of HCl (1 M; ~100 mL). The mixture was filtered and washed with ice cold water (4 × 10 mL). The purple solid was recrystallized by dissolving it in DMF (100 mL), followed by slow addition of water (300 mL). The mixture was filtered and the solid was washed with water (3 × 50 mL). The product was obtained as a purple solid (3.29 g; 4.16 mmol; 6.3%). ^1H NMR (300 MHz, DMSO) δ 13.26 (s (br), 4H), 8.86 (s, 8H), 8.43 – 8.28 (m, 16H), -2.94 (s, 2H).

Fe(5,10,15,20-(4-carboxyphenyl)-porphyrin)chloride (FeTCPP)

TCPP (300 mg; 0.38 mmol) and $FeCl_2$•$4H_2O$ (1.06 g; 5.3 mmol) were dissolved in degassed DMF (40 mL) and refluxed for 5.5 h under N_2 atmosphere. After cooling down to r.t., water (60 mL) was added dropwise. The mixture was filtered and washed with water (3 × 15 mL). The product was obtained as a brown solid (237.7 mg; 0.27 mmol; 71%). Elemental analysis: meas. 65.18 %C, 3.11 %H, 6.31 %N, calc. for [$C_{48}H_{28}ClFeN_4O_8$]: 65.51 %C, 3.21 %H, 6.37 %N.

dPCN-224(Fe)

*d*PCN-224(Fe) was made by modifying a literature procedure.[2] $ZrCl_4$ (37.5 mg, 0.16 mmol), FeTCPP (25 mg; 0.03 mmol) and benzoic acid (1.35 g) were suspended in DMF (4 mL) and sonicated until dissolved (20 min) in a 20 mL screw-cap scintillation vial (PerkinElmer). The brown solution was stirred with a stirrer bar (PTFE, 10 × 5 mm, VWR) at 400 rpm in a pre-heated 120 °C oil bath for 24 h. The resulting suspension was poured in a 15 mL polypropylene centrifuge tube (VWR) and was centrifuged at 4000 rpm for 20 min. The supernatant was discarded and the residue was soaked in fresh DMF (14 mL on the tube) twice for 2 h, with the same centrifugation/re-suspension step in between. The solid was activated by dispersing it in 10 mL DMF with 0.5 mL HCl (8 M in water) in a 20 mL screw-cap scintillation vial. The mixture was heated overnight in a 100 °C oven. After cooling to r.t., the suspension was poured in a 15 mL polypropylene centrifuge tube and consecutive DMF (2 × 2 h) and acetone washing cycles (4 × 24 h) were performed following the same centrifugation/re-suspension procedure. The final material was dried in a 60°C vacuum oven overnight to yield a brown solid (14 mg).

1.3 Electrochemistry

Electrochemical set-up

All electrochemical measurements were conducted in custom-made 2-compartment cells (50 mL) in which the counter electrode is separated from the main compartment *via* a glass frit. Moreover, the reference electrode was separated from the main compartment *via* a Luggin capillary. All glassware used for electrochemical measurements was routinely cleaned from organic materials by overnight soaking in a solution of $KMnO_4$ (1 g/L) in H_2SO_4 (0.5 M) followed by a 30 min immersion into water containing a few droplets of concentrated H_2SO_4 and H_2O_2 to remove any manganese traces. Afterward, the glassware was boiled three times for 40 min in water. Milli-Q Ultrapure grade water (>18.2 MΩ cm resistivity) was used for cleaning of the electrochemical cells. The experiments were performed using a 3-electrode set-up equipped with an Autolab PGSTAT 12 Potentiostat with a MSR rotator (Pine instrument) and operated under NOVA software. All electrochemical experiments were conducted under an atmosphere of oxygen (Linde, O_2 5.0) or argon (Linde, Ar 5.0). Prior to each experiment, the respective gas was bubbled through the electrolyte solution for at least 15 min. During the measurements, the gas was allowed to continue bubbling in the electrolyte to obtain a stable dissolution of the gas during measurements. For all measurements, a 0.15 M HNO_3 and 0.15 M $NaNO_3$ was used made with HNO_3 (Suprapur, 65%) and $NaNO_3$ (Suprapur, 99.99%) dissolved in Milli-Q Ultrapure grade water (>18.2 MΩ cm resistivity).

As working electrode, a glassy carbon (GC) disk electrode (d = 5 mm, Pine Instrument) was used in combination with a ChangeDisk Peek electrode holder (E5TQPK, Pine Instrument). The GC electrode was polished on a Struers LaboPol-30 polishing machine by rotating the polishing cloth (Dur-type) at 200 rpm clockwise and moving the electrode in a counterclockwise circular motion over the pad. The GC electrode was first polished with a diamond suspension (1.0 µm, DiaPro, Struers) on the polishing cloth for 1 min, after which the electrode was rinsed with isopropanol and water. Next, the electrode was polished on a different polishing cloth with a silica suspension (OPS Non-dry, Struers) for 1 min, after which the electrode was rinsed with water and sonicated in water for 10 minutes. For the rotating ring disk electrode (RRDE) experiments the platinum ring was also manually polished for 4 min with the diamond suspension and for 2 min with the silica suspension. As counter electrode, a coiled Au wire (MaTeck, 0.6 mm) was used. This Au coil was flamed and rinsed with water prior to each measurement. As reference electrode a Pt gauze was used while bubbling H_2 (Linde, H_2 5.0) through the buffer solution in the Luggin capillary. Fluorine doped tin oxide (FTO) electrodes (7Ω, Merck) were manually cut to plates of 2.5 × 0.5 cm with a diamond cutter. The FTO plates were cleaned by successive sonication in soap (Liquinox), water, isopropanol, ethanol, and acetone (15 min each), after which the electrode was dried overnight in a vacuum oven (40 °C).

Catalyst ink preparation

For the catalyst ink preparation, Nafion (Merck, 5 wt% in alcohols), carbon black (CB, Alpha Aesar, acetylene 50% compressed), and acetone (Merck) were used. For *d*PCN-224(Fe), the ink was made by combining *d*PCN-224(Fe) (1.5 mg), CB (1.5 mg), Nafion (20 µL) and acetone (180 µL) in a 1.5 mL autosampler vial. For FeTCPP, the ink consisted of FeTCPP (0.9 mg), CB (1.5 mg), Nafion (20 µL) and acetone (180 µL), which results in the same iron loading of 1.1 µmol in both inks. The ink mixture was sonicated for 20 min to allow mixing. Prior to dropcasting the ink on the electrode, the mixture was placed on a vortex for 10 seconds to allow for maximum homogeneity in the suspension. Before the ink was dropcasted on the electrode, the cleanliness of the GC electrode was verified by measuring a rotating disk electrode cyclic voltammogram (RDE CV). Once it has been established that the carbon electrode behaved as expected, the electrode was rinsed with water and dried with a precision wipe (Kimtech

Science), after which 10 µL of the ink was dropcasted on the electrode. The dropcast was allowed to dry for at least 15 min in air, after which the measurements were carried out.

Rotating (ring) disk electrode cyclic voltammetry

Rotating (ring) disk electrode cyclic voltammetry (R(R)DE CV) measurements were conducted with a scan rate of 50 mV/s, step size of 5 mV, and rotation rate of 1600 rpm. The faradaic efficiency towards H_2O_2 ($\%H_2O_2$) was determined according to the following equation:

$$\%H_2O_2 = \frac{2\ x\ (i_{ring}\ /\ N_{H_2O_2})}{i_{disk} + (i_{ring}\ /\ N_{H_2O_2})} \times 100\%$$

With i_{ring} being the current on the ring, i_{disk} the current on the disk, and $N_{H_2O_2}$ the collection efficiency of H_2O_2 on the ring. $N_{H_2O_2}$ was determined prior to each measurement by measuring a GC blank RRDE LSV between 1.0 and -0.5 V vs. RHE and dividing the current on the ring by the current on the disk. Here it is used that the GC electrode can only perform the 2-electron reduction of oxygen towards hydrogen peroxide.[3] The average of the collection efficiency between -0.3 and -0.5 V vs. RHE was used as collection efficiency for the measurement with catalyst ink.

2. N_2-isotherm *d*PCN-224(Fe)

To obtain a N_2-isotherm of *d*PCN-224(Fe), the powder was dried for 12 h at 80°C under vacuum as pretreatment. The N_2-isotherm is shown in Figure S1 and the data was analyzed with BELMaster software to calculate the pore size distribution with the Mikhail-Brunauer micropore (MP) and Barrett-Joyner-Halenda (BJH) methods. Analysis of the N_2-isotherm indicated pore sizes of 0.8 and 2.0-2.5 Å, as expected for PCN-224.[4] Combined these analytical data for the MOF sample are in good agreement with a *d*PCN-224 sample of good crystallinity.[5]

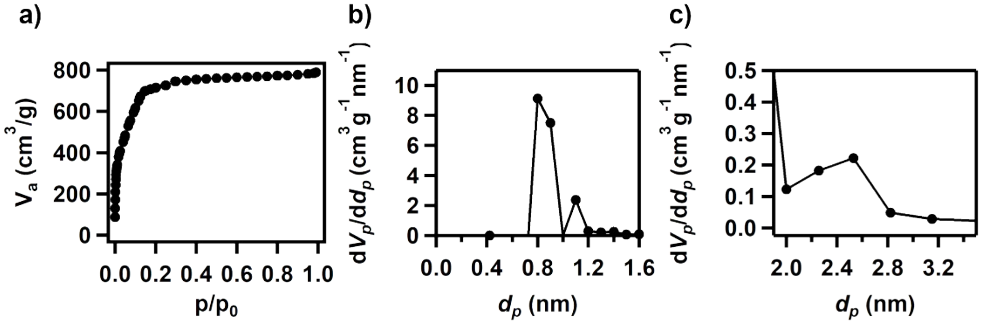

Figure S1. (a) N_2 adsorption isotherm of *d*PCN-224(Fe) at 77 K, 1 atm. (b) MP and (c) BJH pore size distribution fitting of the N_2 isotherm of *d*PCN-224(Fe).

3. PCN-224(Fe) chemical stability in electrolyte solution

The stability of PCN-224(Fe) in 0.1 M borate buffer (pH 8.5), acetate buffer (pH 4.7), phosphate buffer (pH 7), NaOH, $NaNO_3$ (pH 7), Na_2SO_4 (pH 5), H_2O_2, and H_2SO_4 was determined by mixing ~1 mg of the MOF with ~10 mL of electrolyte (Figure S2a). Direct dissolution of PCN-224(Fe) was observed in phosphate buffer, borate buffer and NaOH, which indicates the MOF in not stable under alkaline conditions. This chemical stability test indicates that PCN-224(Fe) is chemically stable in acetate buffer, $NaNO_3$, Na_2SO_4, and H_2SO_4 solutions.

Electrochemical cyclic voltammogram (CV) measurements were carried out in 0.1 M electrolyte solutions under oxygen atmosphere. Leaching of the PCN-224(Fe) from the working electrode was seen for acetate buffer, $NaNO_3$, and Na_2SO_4 electrolyte solutions already during the first CV cycle (Figure S2b and S2c). Only acidic electrolytes such as H_2SO_4, HNO_3, and $HClO_4$ resulted in the retention of the MOF on the working electrode.

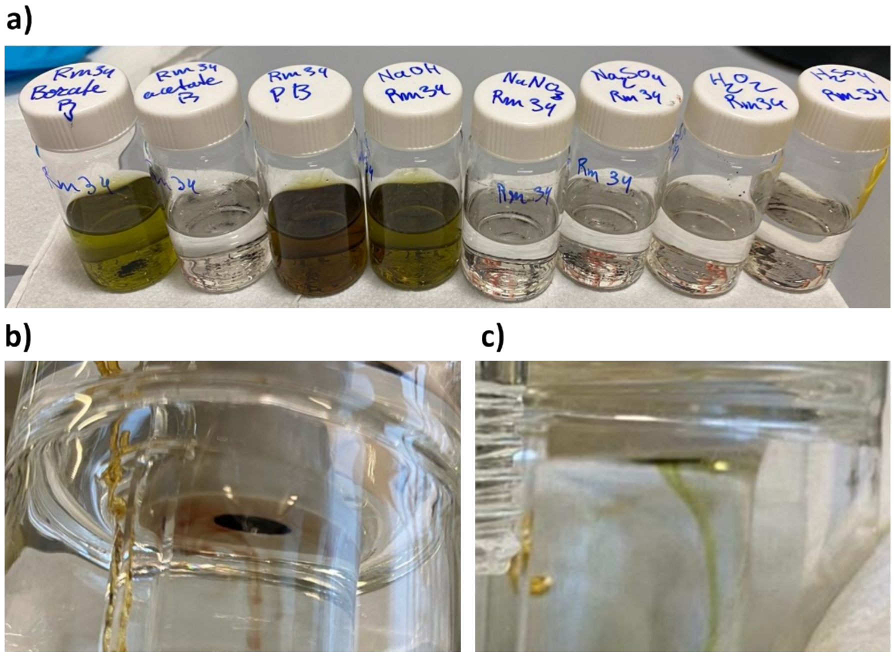

Figure S2. (a) Chemical stability test of PCN-224(Fe) in 0.1 M electrolytes of (from left to right) borate buffer (pH 8.5), acetate buffer (pH 4.7), phosphate buffer (pH 7), NaOH, $NaNO_3$ (pH 7), Na_2SO_4 (pH 5), H_2O_2, and H_2SO_4. Picture of the working electrode during cyclic voltammogram measurement with PCN-224(Fe) in (b) Na_2SO_4 or (c) $NaNO_3$ under oxygen atmosphere.

4. RDE CV with *d*PCN-224(Fe) and FeTCPP

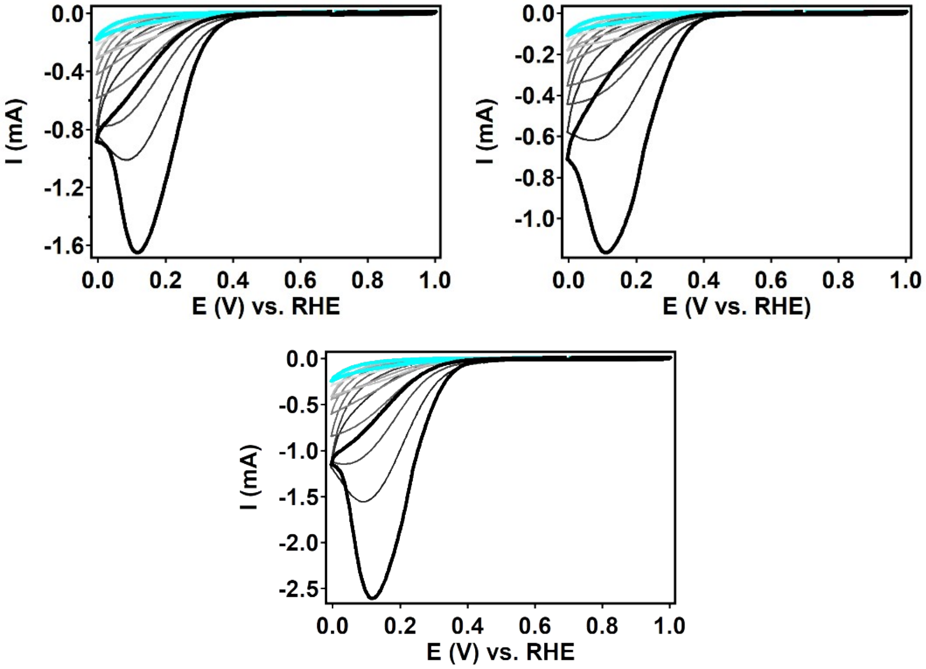

Figure S3. RDE CV with *d*PCN-224(Fe) as catalyst in triplo. The first scan is indicated in black and the ninth scan in teal. Measured in 0.15 M HNO_3 and 0.15 M $NaNO_3$ with 50 mV/s scan rate and 1600 rpm rotation rate under an oxygen atmosphere.

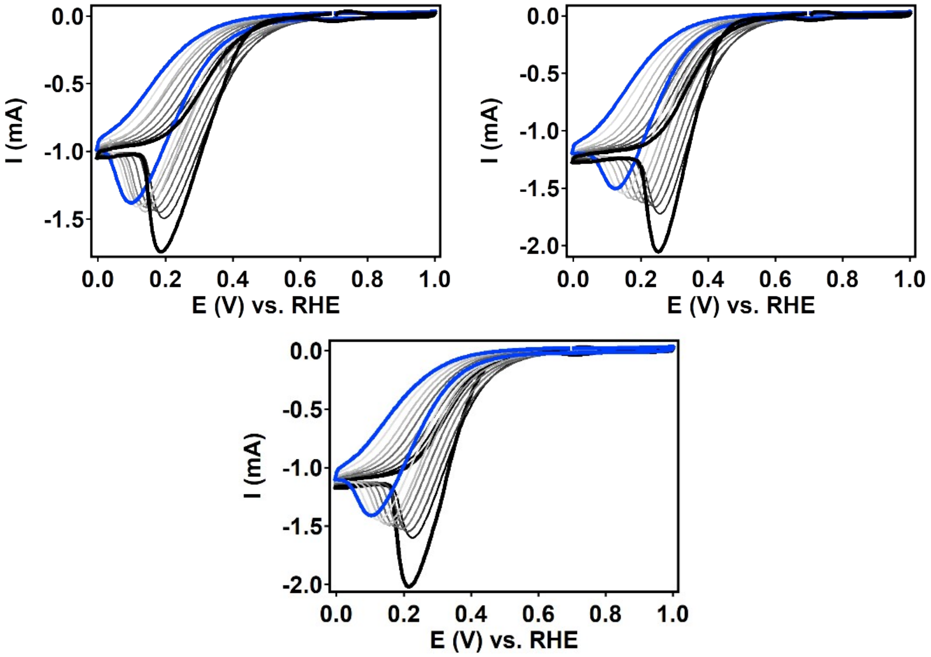

Figure S4. RDE CV with FeTCPP as catalyst in triplo. The first scan is indicated in black and the ninth scan in dark blue. Measured in 0.15 M HNO_3 and 0.15 M $NaNO_3$ with 50 mV/s scan rate and 1600 rpm rotation rate under an oxygen atmosphere.

5. ORR with PCN-224(Fe)

One sample of PCN-224(Fe) was synthesized in which the pXRD showed the reflections at 3.2 and 5.5° that indicate the presence of ordered PCN-224 superstructure domains in the MOF (Figure S5). The reproducibility of the synthesis method proved to be limited and in most synthesis attempts *d*PCN-224(Fe) is obtained instead.[5] Due to the limited amount of PCN-224(Fe) sample obtained, it was decided to carry out the study presented in this chapter with *d*PCN-24(Fe). Nevertheless, with the PCN-224(Fe) sample an RDE CV under oxygen atmosphere was performed in 0.1 M $HClO_4$ with a GC electrode (d = 5 mm) containing a dropcast of 10 µL ink (Figure S6). The ink consisted of 1 mg PCN-224(Fe), 1 mg CB, 180 µL acetone and 20 µL Nafion (5%). The RDE CV shows a decreasing current over multiple scans for PCN-224(Fe) as well as for *d*PCN-224(Fe). This indicates that the structural difference between PCN-224 and dPCN-224 does not influence the ORR catalysis.

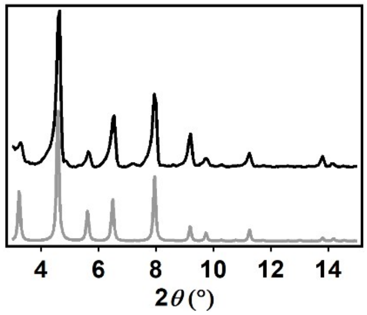

Figure S5. pXRD spectrum of PCN-224(Fe) synthesized in this work measured at 2°/min (black) and the theoretical spectrum of PCN-224 (grey).

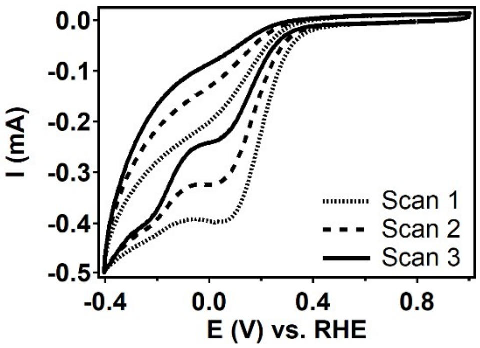

Figure S6. First three scans of RDE CV of PCN-224(Fe). Measured in 0.1 M $HClO_4$ with 50 mV/s scan rate and 1600 rpm rotation rate under an oxygen atmosphere.

6. DPV PCN-224(H_2)

PCN-224(H_2) can be made when TCPP is used as the porphyrin linker without a metal coordinated in the N_4 binding pocket. This PCN-224(H_2) was dropcasted with CB, Nafion and acetone on a GC electrode and a DPV experiment was carried out (Figure S7). A redox peak is seen around 0.6 V vs RHE, which is also present for PCN-224(Fe), and assigned to redox features of carbon black.

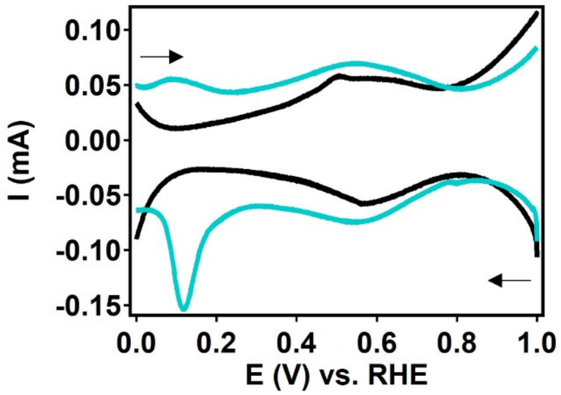

Figure S7. DPV measurement of PCN-224(Fe) (teal) and PCN-224(H_2) (black). Measured in 0.5 M $HClO_4$ and 0.5 M $NaClO_4$ with 1 mV step size, 50 mV modulation amplitude, 3 ms modulation time and 50 ms interval time under an argon atmosphere.

7. RRDE LSV with *d*PCN-224(Fe) and FeTCPP

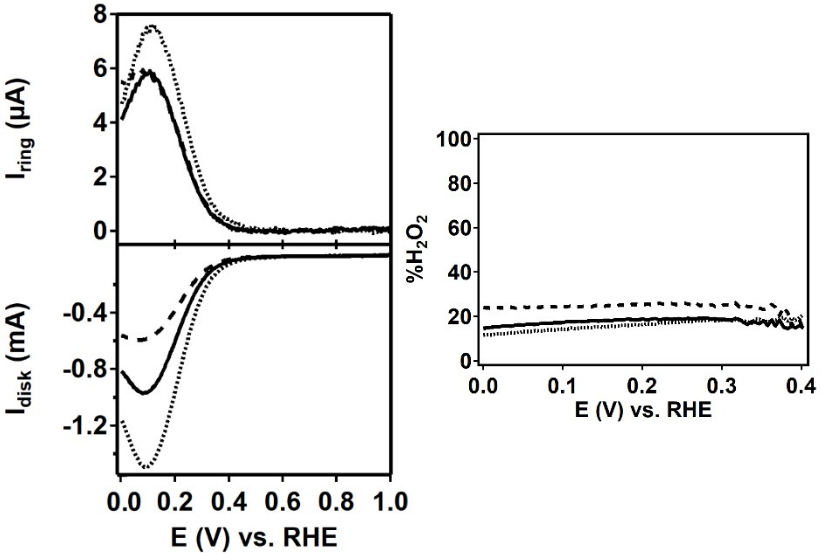

Figure S8. RRDE LSV and %H_2O_2 with *d*PCN-224(Fe) as catalyst in triplo. Measured in 0.15 M HNO_3 and 0.15 M $NaNO_3$ with 50 mV/s scan rate and 1600 rpm rotation rate under an oxygen atmosphere.

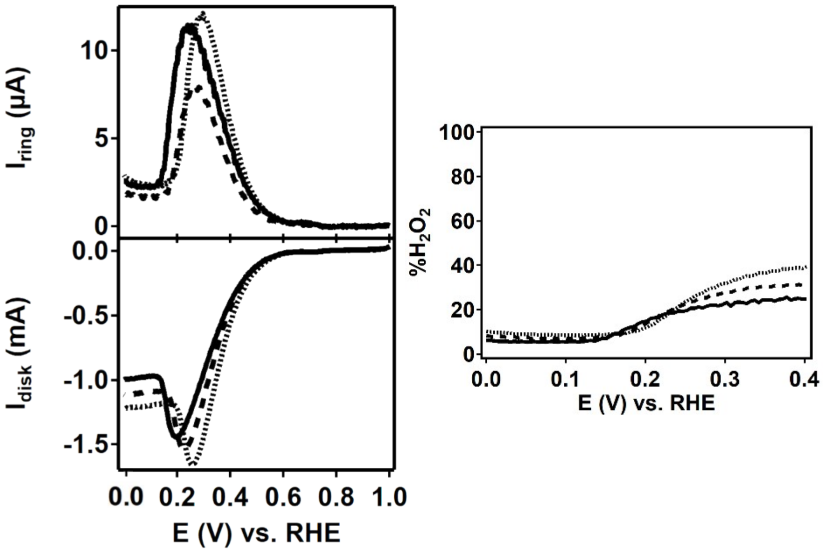

Figure S9. RRDE LSV and %H_2O_2 with FeTCPP as catalyst in triplo. Measured in 0.15 M HNO_3 and 0.15 M $NaNO_3$ with 50 mV/s scan rate and 1600 rpm rotation rate under an oxygen atmosphere.

8. UV-Vis study with PCN-224(Co) and methylene blue

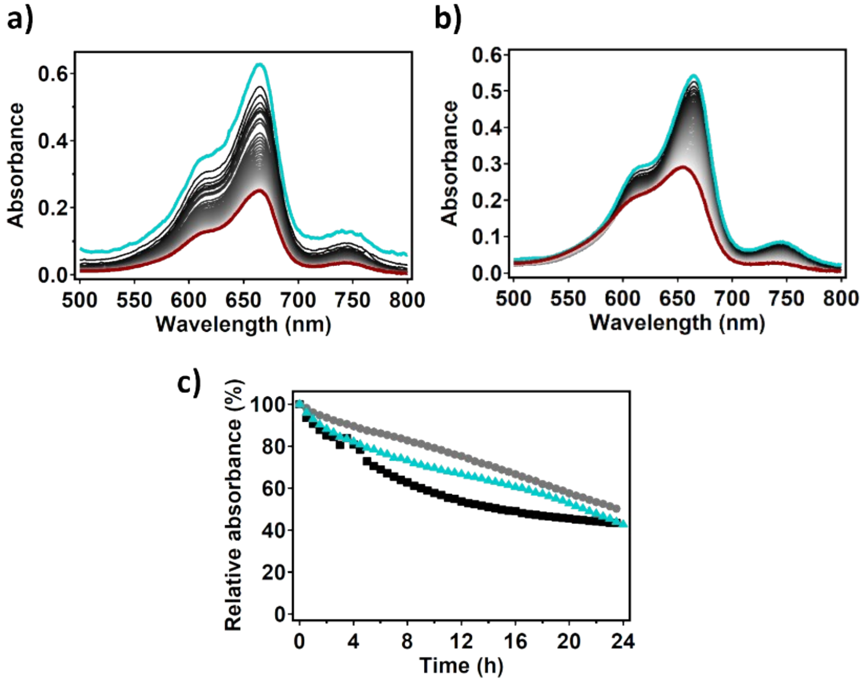

Figure S10. UV-Vis spectra with (a) PCN-224(Co) and MB, and (b) PCN-224(Co), MB and H_2O_2 measured every 30 minutes for 24 h. The spectrum at t=0 is shown in teal and the curve at t = 24 h is shown in red. (c) The relative absorbance at 665 nm over time under different conditions of [PCN-224(Co)+MB] (grey), [PCN-224(Co)+H_2O_2+MB] (black), and [PCN-224(Fe)+MB] (teal). Concentrations: 6.0 µg/mL MB, 10 mM H_2O_2, and 0.17 mg/mL PCN-224(Co) with a total cuvette volume of 3 mL.

9. UV-Vis study with *d*PCN-224(Fe) and methylene blue

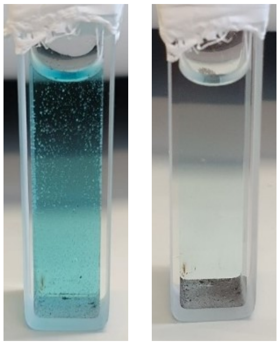

Figure S11. (left) Bubbles formed when H_2O_2 was added to a mixture of PCN-224(Fe) and MB. (right) Mixture after 24 h. Concentrations: 6.0 µg/mL MB, 10 mM H_2O_2, and 0.17 mg/mL PCN-224(Co) with a total cuvette volume of 3 mL.

10. SEM *d*PCN-224(Fe)

*d*PCN-224(Fe) ink containing the catalyst, CB, acetone and Nafion was dropcasted onto two clean FTO electrodes (2.5 × 0.5 cm, 10 μL dropcast). One of these electrodes was used for 20 CV scans under oxygen atmosphere (Figure S12). After the electrocatalysis the electrodes were soaked in water for three days, with refreshing the water each day to remove the electrolyte salts from the pores. To obtain a dry sample after catalysis, the FTO plate with dropcast was soaked in DCM for three days with refreshing of the DCM each day to remove the water molecules. Afterwards, the FTO plate was allowed to dry in air. A fresh *d*PCN-224(Fe) dropcast on FTO and the sample of *d*PCN-224(Fe) on FTO used for ORR catalysis were investigated with SEM measurements (Figure S13).

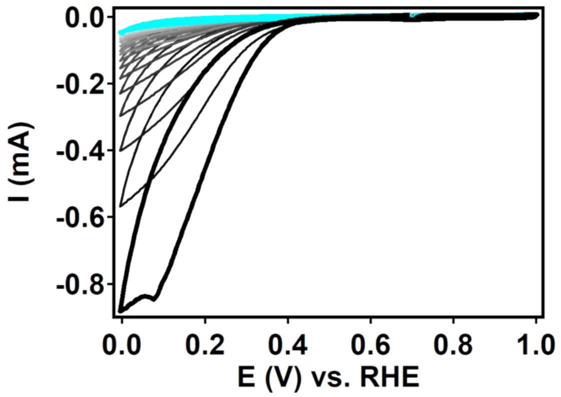

Figure S12. CV measurement of *d*PCN-224(Fe) dropcasted on an FTO electrode. The first scan is indicated in black and the twentieth scan in teal. Measured in 0.15 M HNO_3 and 0.15 M $NaNO_3$ with 50 mV/s scan rate under an oxygen atmosphere.

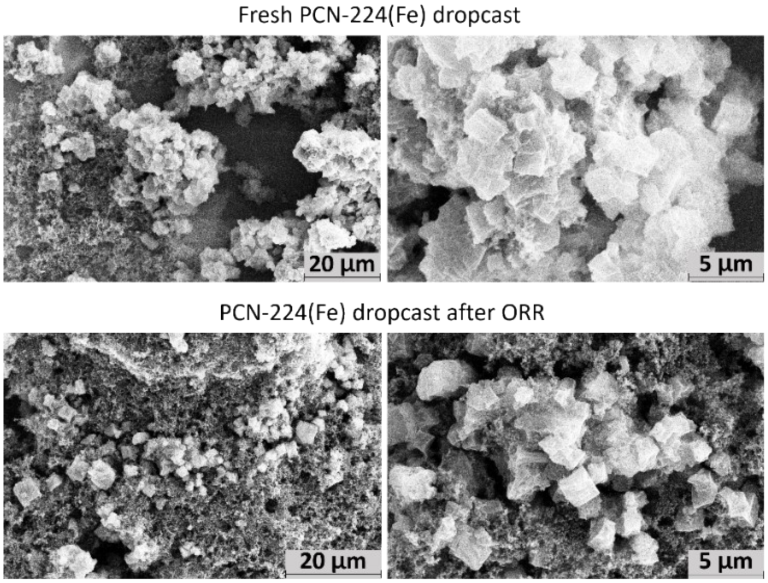

Figure S13. SEM images of (top) an FTO electrode with a fresh dropcast and (bottom) an FTO electrode with a dropcast used for catalysis. Images were measured at 15 kV and 0.1 nA.

11. ICP-MS PCN-224(Fe)

*d*PCN-224(Fe) ink containing the catalyst, CB, acetone and Nafion was dropcasted onto three clean FTO electrodes (2.5 × 0.5 cm, 10 μL dropcast). Two of these electrodes were used for a CV measurement under oxygen atmosphere of twenty scans (Figure S14). After the electrocatalysis the electrodes were soaked in water for three days, with refreshing the water each day to remove the electrolyte salts from the pores. After soaking the electrodes in water, the electrodes were transferred to a clean microwave tube containing HNO_3 (Suprapur 65%, 4 mL) and H_2O_2 (30% w/w, 1 mL) and heated in a 90°C oven for four days. Additionally, a FTO electrode with a fresh dropcast that was not used for electrochemistry was digested. After cooling to r.t., the zirconium and iron contents of the acidic solutions were determined with ICP-MS analysis.

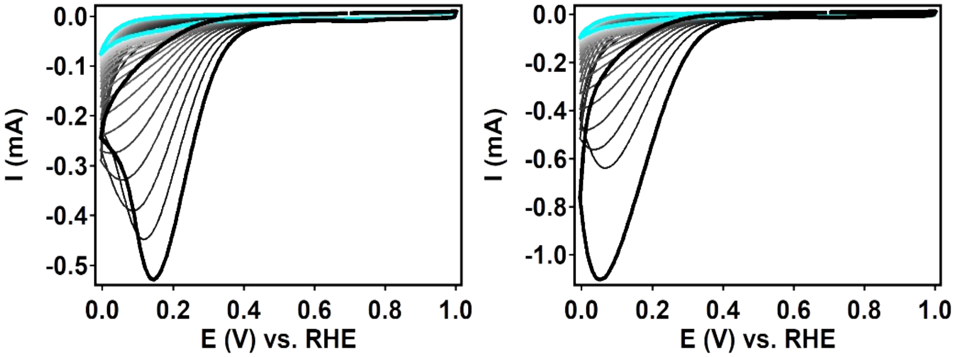

Figure S14. CV measurements of *d*PCN-224(Fe) dropcasted on FTO electrodes. The first scan is indicated in black and the twentieth scan in teal. Measured in 0.15 M HNO_3 and 0.15 M $NaNO_3$ with 50 mV/s scan rate under an oxygen atmosphere.

The ICP-MS data is presented in Table S1. The content determination was carried out in duplo for each sample. In the fresh sample 2.09 ± 0.01 Fe ions per Zr_6 node were found, while the used samples showed 2.02 ± 0.10 Fe centers per node. Therefore, no significant decrease of the iron content *d*PCN-224(Fe) after catalysis is observed.

Table S1. Zirconium and iron content determined with ICP-MS analysis of one fresh *d*PCN-224(Fe) dropcast and two dropcasts that were used for electrocatalysis.

Sample	Zr (10^{-6} g/L)	Fe (10^{-6} g/L)	Zr (mol/L)	Fe (mol/L)	Fe / Zr_6
**Fresh**	30.618	6.392	0.336	0.116	2.080
**Fresh**	30.467	6.412	0.334	0.117	2.097
**Used #1**	29.992	6.526	0.329	0.119	2.168
**Used #1**	31.059	6.190	0.340	0.113	1.986
**Used #2**	27.114	5.381	0.297	0.098	1.977
**Used #2**	27.570	5.417	0.302	0.099	1.958

12. XPS of *d*PCN-224(Fe)

An FTO electrode was covered with 50 µL ink containing *d*PCN-224(Fe), CB, Nafion and acetone and used for 20 CV cycles under oxygen atmosphere (Figure S15). After the electrocatalysis the electrode was soaked in water for three days, with refreshing the water each day to remove the electrolyte salts from the pores. To obtain a dry sample after catalysis, the FTO plate with dropcast was soaked in DCM for three days with refreshing of the DCM each day to remove the water molecules. Afterwards, the FTO plate was allowed to dry in air. A fresh *d*PCN-224(Fe) dropcast on FTO and the sample of *d*PCN-224(Fe) on FTO used for ORR catalysis were investigated with XPS measurements (survey spectra in Figure S16 and region scans in Figure S17).

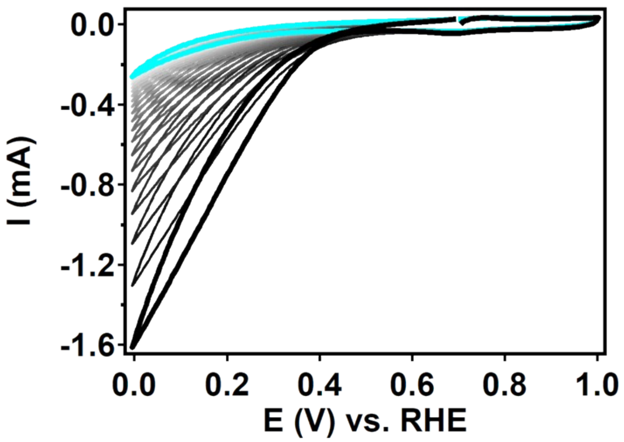

Figure S15. CV measurement of *d*PCN-224(Fe) dropcasted on an FTO electrode. The first scan is indicated in black and the twentieth scan in teal. Measured in 0.15 M HNO_3 and 0.15 M $NaNO_3$ with 50 mV/s scan rate under an oxygen atmosphere.

The survey spectra indicate that the major surface components of both dropcasts consist mainly of oxygen, carbon, and fluorine that originate from the Nafion and CB in the dropcast ink (Figure S16). Sn species seen in the samples indicate low film thickness of the dropcasted layer and are related to the FTO substrate. Region scans were carried out for C s1, Zr 3d, N 1s, Fe 2p, and Fe 3p (Figure S17). The C s1 signal remains constant before and after catalysis with two main peaks at 291.7 and 286 eV, which represent the CF_2 carbons of Nafion and sp^2 carbon of carbon black, respectively.[6, 7] The Zr 3d signal is present before and after catalysis and consists of two peaks at 186.3 and 183.9 eV before catalysis which agrees with earlier reports for PCN-224.[8] The N 1s signal coming from the porphyrin N_4 binding pocket in *d*PCN-224(Fe) can be convoluted into peaks at 401.7 and 399.7 eV before catalysis and at 402.0 and 399.8 eV, which indicates that the porphyrin N_4 pocket remains present at the MOF surface after catalysis. The Fe 2p signal overlaps with the large F 1s signal and cannot be accurately fitted. The absence of a clear Fe 3p signal indicates that the iron content in the dropcast is too low for accurate XPS analysis of iron in the *d*PCN-224(Fe) MOF.

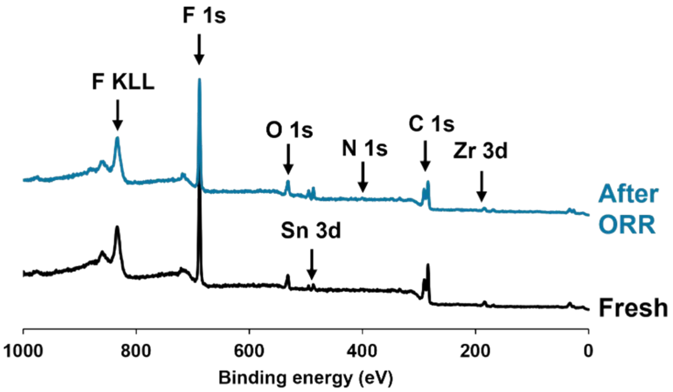

Figure S16. A survey XPS spectrum of *d*PCN-224(Fe) freshly dropcasted in the electrode (black, bottom) and after 20 CV scans of ORR catalysis (teal, top).

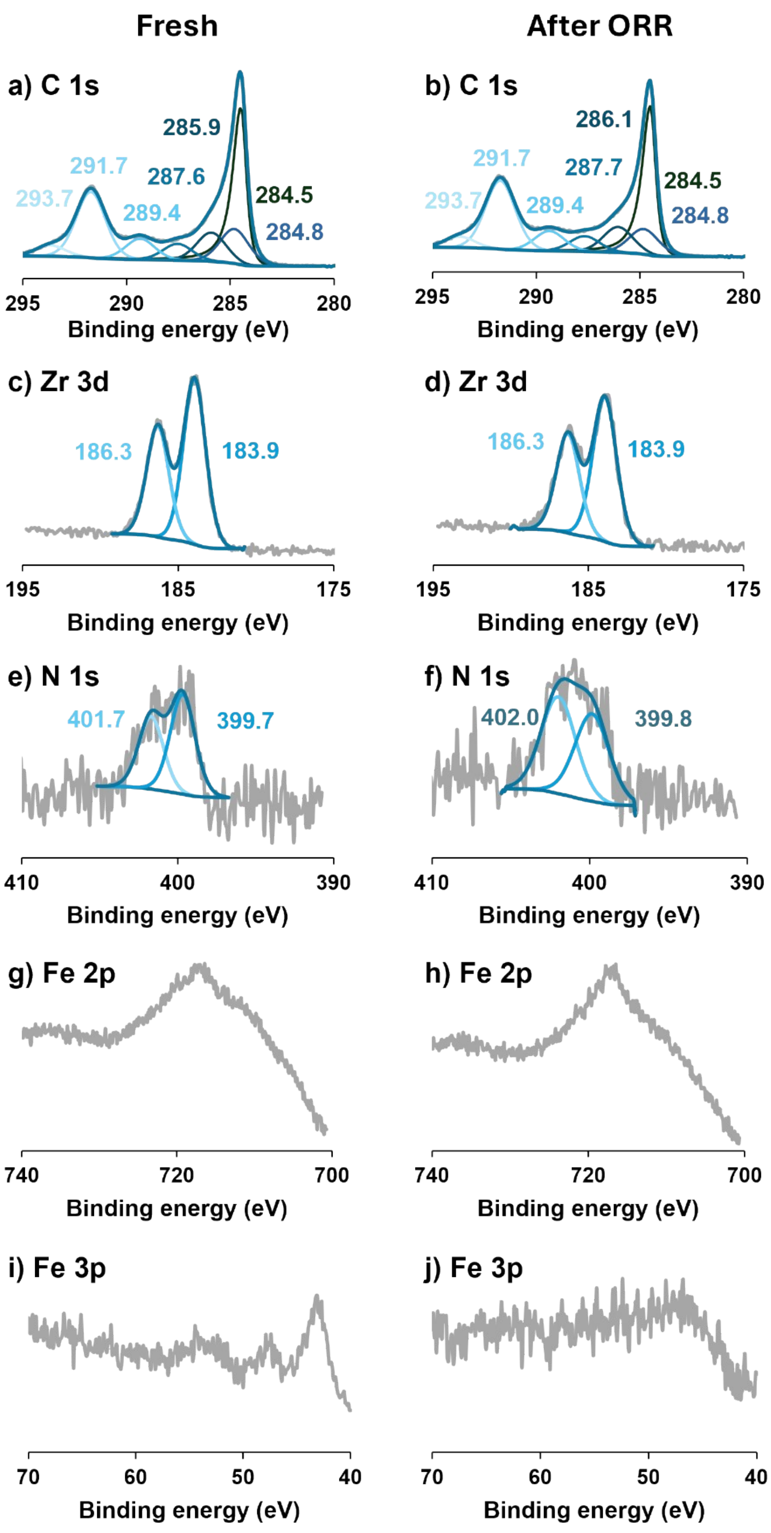

Figure S17. (a, b) C 1s, (c, d) Zr 3d, (e, f) N 1s, (g, h) Fe 2p, and (I, j) Fe 3p XPS spectra of (a, c, e, g) a fresh *d*PCN-224(Fe) dropcast on FTO and (b, d, f, h) after 20 CV scans of ORR catalysis.

13. FTIR of *d*PCN-224(Fe)

A *d*PCN-224(Fe) ink was made without CB, because its presence would prevent light transmission through the sample. The FTO electrode was used for 20 CV cycles under oxygen atmosphere (Figure S18). FTIR spectra were measured for FTO electrodes containing *d*PCN-224(Fe) ink that consisted of the MOF and Nafion in acetone before and after ORR catalysis. To account for the peaks that are due to the Nafion in the dropcast, an FTIR spectrum was also measured for an FTO electrode with a dropcast of Nafion (Figure S19). The signals for Nafion and *d*PCN-224(Fe) are assigned in Table S2 and Table S3, respectively.

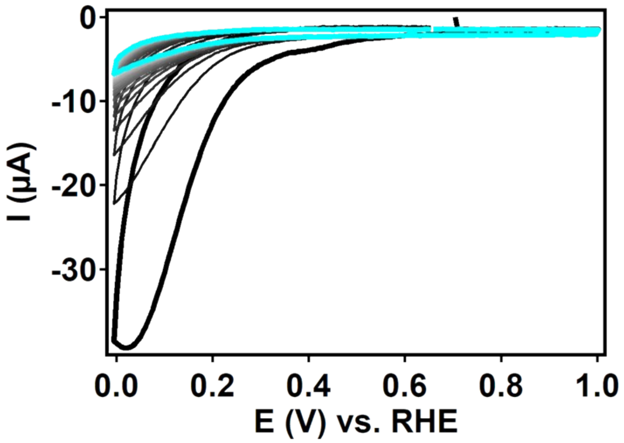

Figure S18. CV measurement of *d*PCN-224(Fe) dropcasted without carbon black on an FTO electrode. The first scan is indicated in black and the twentieth scan in teal. Measured in 0.15 M HNO_3 and 0.15 M $NaNO_3$ with 50 mV/s scan rate under an oxygen atmosphere.

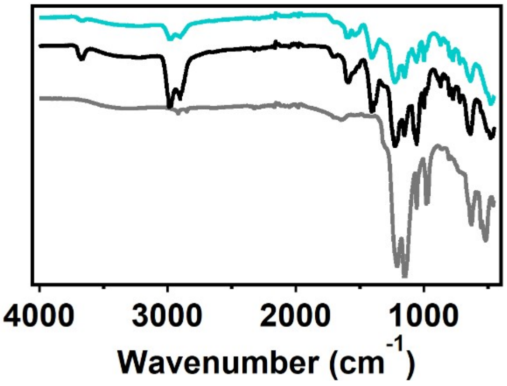

Figure S19. FTIR spectra of FTO with Nafion (grey), a fresh dropcast of *d*PCN-224(Fe) on FTO (black), and a dropcast of *d*PCN-224(Fe) used for electrocatalysis (teal).

Table S2. Assignment of FTIR signals of Nafion and FTO.[9]

Absorption peak (cm^{-1})	Assignment
**520**	C-F
**631**	C-H
**983**	C-O-C
**1057**	S-O
**1150**	$C-F_2$
**1210**	$C-F_2$

Table S3. Assignment of FTIR signals of *d*PCN-224(Fe).[10-12]

Absorption peak (cm^{-1})	Assignment
**711 - 744**	Zr-O, Zr-OH, $ZrOH_2$
**774 - 870**	C-H
**999**	Fe-N
**1408**	C-N, C-O
**1542**	C=N
**1595**	C=C, O-C-O
**1703**	C=O
**2906**	C-H
**2984**	O-H
**3674**	O-H

14. References

1 P. M. Usov, S. R. Ahrenholtz, W. A. Maza, B. Stratakes, C. C. Epley, M. C. Kessinger, J. Zhu and A. J. Morris, *J. Mater. Chem. A* 2016, **4**, 16818.

2 D. W. Feng, Z. Y. Gu, J. R. Li, H. L. Jiang, Z. W. Wei and H. C. Zhou, *Angew. Chem. Int. Ed.* 2012, **51**, 10307.

3 M. Langerman, D. G. H. Hetterscheid, *Angew. Chem. Int. Ed.* 2019, **58**, 12974.

4 D. W. Feng, W. C. Chung, Z. W. Wei, Z. Y. Gu, H. L. Jiang, Y. P. Chen, D. J. Darensbourg and H. C. Zhou, *J. Am. Chem. Soc.* 2013, **135**, 17105.

5 H. L. B. Bostrom, S. Emmerling, F. Heck, C. Koschnick, A. J. Jones, M. J. Cliffe, R. Al Natour, M. Bonneau, V. Guillerm, O. Shekhah, M. Eddaoudi, J. Lopez-Cabrelles, S. Furukawa, M. Romero-Muñiz, Y. Xiong, A. E. Platero-Prats, J. Roth, W. L. Queen, K. S. Mertin, D. E. Schier, N. R. Champness, H. H.-M Yeung and B. V. Lotsch, *Adv. Mater*. 2023, **36**, e2304832. .

6 A. K. Friedman, W. Q. Shi, Y. Losovyj, A. R. Siedle and L. A. Baker, *J. Electrochem. Soc.* 2018, **165**, 733.

7 D. Pantea, H. Darmstadt, S. Kaliaguine and C Roy, *Appl. Surf. Sci.* 2003, **217**, 181.

8 J. H. He, Y. J. Zhang, J. He, X. L. Zeng, X. D. Hou and Z Long, *Chem. Commun.* 2018, **54**, 8610.

9 Z. X. Liang, W. M. Chen, J. G. Liu, S. L. Wang, Z. H. Zhou, W. Z. Li, G. Q. Sun and Q Xin, *J. Membr. Sci.* 2004, **233**, 39.

10 L. Shi, L. Q. Yang, H. B. Zhang, K. Chang, G. X. Zhao, T. Kako and J. H. Ye, *Appl. Catal. B Environ.* 2018, **224**, 60.

11 Y. Q. Zong, S. S. Ma, J. M. Gao, M. J. Xu, J. J. Xue and M. X. Wang, *ACS Omega* 2021, **6**, 17228.

12 S. Carrasco, G. Orcajo, F. Martinez, I. Imaz, D. Arenas-Esteban, D. Maspoch, S. Bals, G. Calleja and P. Horcajada, *Mater. Today Adv.* 2023, **19**, 100930
